# The RNA-binding protein PRRC2B preserves 5′ TOP mRNA during starvation to maintain ribosome biogenesis during nutrient recovery

**DOI:** 10.1093/nar/gkaf1334

**Published:** 2025-12-12

**Authors:** Nadav Goldberg, Doron Bril, Miriam Eisenstein, Tsviya Olender, Alon Savidor, Shani Bialik, Shmuel Pietrokovski, Adi Kimchi

**Affiliations:** Department of Molecular Genetics, Weizmann Institute of Science, Rehovot 7610001, Israel; Department of Molecular Genetics, Weizmann Institute of Science, Rehovot 7610001, Israel; Department of Molecular Genetics, Weizmann Institute of Science, Rehovot 7610001, Israel; Department of Molecular Genetics, Weizmann Institute of Science, Rehovot 7610001, Israel; The de Botton Institute for Protein Profiling of the Nancy and Stephen Grand Israel National Center for Personalized Medicine (G-INCPM), Weizmann Institute of Science, Rehovot 7610001, Israel; Department of Molecular Genetics, Weizmann Institute of Science, Rehovot 7610001, Israel; Department of Molecular Genetics, Weizmann Institute of Science, Rehovot 7610001, Israel; Department of Molecular Genetics, Weizmann Institute of Science, Rehovot 7610001, Israel

## Abstract

PRRC2B is an intrinsically disordered RNA-binding protein that is part of the cell’s translation machinery. Here, we show that PRRC2B has two alternatively spliced mRNA transcripts producing major long and minor short isoforms. Mass spectrometry-based interaction studies indicated that both isoforms associate with the 40S ribosomal subunit and translation initiation factors. Importantly, the long isoform also interacted with additional RNA-binding proteins through its unique Arg/Gly-rich region. Among these is LARP1, a regulator of 5′ terminal oligopyrimidine (TOP) mRNAs under conditions of mTOR inhibition. We discovered that, like LARP1, PRRC2B-long isoform binds to 5′ TOP mRNAs. Moreover, it is necessary for the post-transcriptional preservation of their mRNA levels, particularly those encoding ribosomal proteins, during amino acid starvation. In its absence, the rapid *de novo* translation of ribosomal proteins that takes place upon nutrient recovery is impeded. Overall, our study elucidates a newly discovered function for PRRC2B as an RNA-binding protein that regulates ribosomal biogenesis upon metabolic shift, in addition to its established function in initiating translation of specific mRNA targets.

## Graphical Abstract

**Graphical Abstract:**
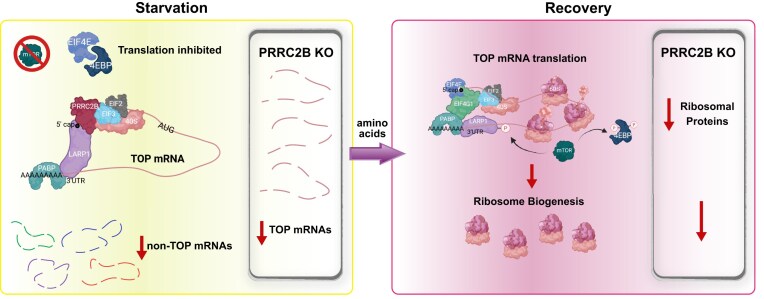

## Introduction

RNA-binding proteins (RBPs) are important regulators of gene expression and homeostasis. These proteins affect and accompany every step of the mRNA life cycle, from transcription, splicing, and nuclear export, to localization, translation, stability, and degradation [1]. Their functions are important in normal growth conditions, and following various stresses, such as nutrient starvation, oxidative stress, and DNA damage [2, 3]. RBPs are also crucial for embryonic development, pluripotency, differentiation, and tumorigenesis [4]. The RNA interactome is now recognized as consisting of thousands of proteins, thanks to large-scale mass spectrometry-based proteomic screens of RNA-interacting proteins. This, combined with techniques involving UV-crosslinking and RNA sequencing, has uncovered both the target mRNAs and the interacting interface of many RBPs, greatly advancing our understanding of RNA-protein regulatory processes [5].

Proline-rich coiled-coil 2 (PRRC2) proteins are a family of RBPs comprised of paralogues PRRC2A, B, and C, all of which are large intrinsically disordered proteins. All three were identified in unbiased interactome screens for RBPs [6–12]. PRRC2A has been shown to be an m(6)A reader that can stabilize its target mRNA, Olig2, through binding a methylated consensus motif, thereby ultimately regulating oligodendrocyte development [13]. This property is shared by the family, and PRRC2B similarly stabilizes *SOX2* mRNA to facilitate oligodendrocyte progenitor cell development and myelination [14]. In endothelial cells, PRRC2B also binds m(6) A-containing mRNAs and regulates the expression of ECM components by mechanisms involving mRNA splicing and mRNA decay [15]. In addition, all 3 PRRC2 proteins localize to stress granules, and PRRC2C was shown to be necessary for stress granule formation [16,  17,]. Notably, recent data is consistent with a role in translation initiation, as the PRRC2 proteins associate with translation initiation factors and the pre-initiation complex (PIC) and fractionate with both 40S ribosome and 43–48S PICs [18]. Deletion of PRRC2 proteins from HeLa cells leads to decreased global translation rates and a corresponding decrease in proliferation [18]. The phenotype is more severe with double or triple deletions/depletion, suggesting either redundant functions within the family or a combined effect of the loss of specific targets of each family member. Ribosome footprinting of the triple KD cells indicated that PRRC2 proteins direct the translation of specific mRNA targets with long 5′UTRs that harbor upstream reading frames (uORFs). Moreover, PRRC2C selectively binds ribosomes on mRNAs with uORFs, correlating with those mRNAs that show decreased translation efficiency upon PRRC2 knockdown. Furthermore, PRRC2 was shown to promote leaky scanning through these uORFs, thereby driving translation of the main ORF [18]. The *Drosophila* PRRC2 homologue, Nocte, was similarly shown to direct translation of mRNA targets with uORFs, but this was shown to be promoted by re-initiation downstream to the uORFs [19]. PRRC2B was shown to bind mostly to the 5′ UTR of its target mRNAs, within proximity of the translation start site, specifically recognizing GA and CU-rich motifs [20]. mRNAs whose translation requires PRRC2B include oncogenes and cell cycle regulators, specifically, cyclin D [20]. While these studies have significantly advanced our understanding of PRRC2 function, the disparate functions elucidated so far imply multiple roles for these proteins, and additional mRNA targets and RNA-regulatory functions remain to be discovered.

In this study, we focus on PRRC2B and shed new light on its role as an RNA-regulatory protein. We identified two splice isoforms, a long and short form, and assessed their respective functions. While both forms interact with translation initiation factors and the 40S ribosome, the long form has a unique repertoire of interacting proteins that confer additional functions. Specifically, PRRC2B_L_ interacts with LARP1 and PABP proteins and binds to 5′TOP mRNAs. PRRC2B is involved in the preferential preservation of TOP mRNA levels, in particular those encoding ribosomal proteins, during prolonged amino acid starvation, in a post-transcriptional manner. The depletion of PRRC2B during starvation prevents the maintenance of these mRNAs, resulting in inhibition of *de novo* translation of ribosomal proteins upon refeeding that may slow down the recovery of cells from metabolic stress. Thus, we have identified a new function for PRRC2B that designates it as a player within the complex mechanism regulating TOP mRNAs during mTOR inhibition.

## Materials and methods

### Reagents

Antibodies used in this study are as follows: Primary antibodies: HA (Biolegend, cat# 901 501), PRRC2B (Santa Cruz Biotechnology, cat# sc-393604), tubulin (Sigma, cat# T9026T9026), vinculin (Sigma, cat# V9131), EIF3D (Bethyl, cat# A301-758A), EIF4G2 (BD Bioscience, cat# 610 742), LARP1 (Proteintech, cat# 13708–1-AP), PABP (Santa Cruz Biotechnology, cat# sc-32318), PRMT1 (Cell signaling, cat# 2449S), Calnexin (Abcam, cat# ab22595), RPS3 (Proteintech, cat# 66046–1-Ig), RPS6 (Santa Cruz Biotechnology, cat# sc-74459). Secondary antibodies were either horseradish peroxidase (HRP)-conjugated goat anti-mouse (Jackson ImmunoResearch Labs, cat# 115–035-003) or anti-rabbit (Jackson ImmunoResearch, cat# 111–165-144). For IP, anti-HA beads (Sigma, cat# A2095).

Kits used in this study are as follows: Monarch total RNA miniprep kit (NEB, cat # T2010S); Dynabeads™ mRNA DIRECT™ Purification Kit (Invitrogen, cat# 61 012); AzuraQuant™ II cDNA Synthesis Kit (AZ-2504); KAPA HiFi HotStart Ready Mix PCR Kit (Roche, cat# 07 958 927 001); QIAquick PCR Purification Kit (Qiagen, cat# 28 104); AzuraView™ GreenFast qPCR Blue Mix LR (AZ-2305); Pierce™ BCA Protein Assay (Thermo Scientific, cat# 23 227); Click-iT protein reaction kit (Invitrogen, cat# C10276); SuperSignal™ West Pico PLUS Chemiluminescent Substrate (Thermo Scientific, cat# 34 580); TruSeq Stranded mRNA-seq kit (Illumina, cat# 20 020 595).

Enzymes used in this study are as follows: Reverse polymerase SuperScript III (Invitrogen, cat# 18 080 044); Restriction enzymes KpnI, EcorV, DpnI (NEB, cat# R3142S, R3195S, R0176S).

Instruments used included: for fluorescence microscopy, a LSM900 confocal microscope (Zeiss); for RNA quantitation, Qubit (Thermo Fisher scientific) or TapeStation (Agilent); for quantitative real time PCR (qRT-PCR), QuantStudio™ 5 Real-Time PCR System; for RNA deep sequencing, Illumina Novaseq Plus X 1.5B 300 cycles (Illumina); for LC-MS,10 kpsi nanoAcquity Ultra Performance Liquid Chromatography (Waters, Milford, MA, USA), mass spectrometer (Thermo Scientific), and Nanospray Flex Ion Source (Proxeon).

### Biological Resources, cell culture, and transfections

HEK 293T cells (ATCC, cat# CRL-3216) were maintained in Dulbecco’s Modified Eagle’s Medium (DMEM; Sartorius cat# 01–055-1A) supplemented with 2 mM glutamine (Gibco BRL), 100U/ml penicillin and streptomycin (Gibco BRL), and 10% fetal bovine serum (Gibco BRL). For starvation experiments, cells were washed in PBS and grown for 48 h in EBSS media (Sartorius cat# 02–010-1A). Starved cells were treated for 24 h with 50 nM actinomycin D (Sigma-Aldrich, cat# A1410) to avoid toxicity of combined treatment. When indicated, cells were treated for 4 h with 1 µM thapsigargin (Sigma-Aldrich T9033), 100 µM sodium arsenite, or 500 µM cobalt chloride (Sigma-Aldrich, 15 862). U2OS cells expressing GFP-G3BP [21] were obtained from Prof. Eran Hornstein (Weizmann Institute) and grown in DMEM. Additional cell lines used for Western blot expression analysis were grown in DMEM. Cell lines were routinely tested for mycoplasma contamination and, when relevant, authenticated by STR profiling.

Plasmids for expression of exogenous proteins were introduced to cells by the standard calcium-phosphate transfection method or Lipofectamine 2000 transfection reagent (Invitrogen) according to the manufacturer’s protocol. Transient PRRC2B KD was generated by transfecting HEK293T cells with control siRNA (Dharmacon D-001810–10) or siRNA targeting PRRC2B (Dharmacon L-032577–01-0005) using Lipofectamine 2000. Sequences targeting both isoforms: 5'-TGAATGACCAAGACGGAAA-3' (CDS), 5'-AGTGTAAGCAGGCACGAAA-3' (CDS), 5'-CCACACAGCTCATCGTGAA-3′ (3' UTR). Sequence of siRNA targeting PRRC2B_L_ only (exon 16): 5′-GCTGAGCAATTGCGGGTAT-3′.

### Generation of PRRC2B KO Cells

PRRC2B KO HEK 293T cells were generated using lentiviral-mediated CRISPR-CAS9 as previously described [22]. Briefly, guides targeting a region in exon 2 that contains the start codon (Guide 1: 5′-TTTCAAAGGCAGATCGGGAG-3′, Guide 2: 5′-GTAGACGCGATTAGATCCTC-3′) were cloned into lentcrispr V2 vectors carrying CAS9 and puromycin resistance genes. The guides target both isoforms as the *N*-terminus is shared. A non-targeting guide (5′-TTTCGTGCCGATGTAACA-3′) was used for control KO. Virions were generated by standard methods. HEK 293T cells were infected, followed by selection with 1.5μg/ml Puromycin for 6 days. Genotyping was performed after selection using the following primers: Forward Primer 1: 5′-GTACTCCACAGATCGCCTCG-3′, Reverse Primer 2: 5′-TACATGCACACCCAGAAGCC-3′. Pools of infected cells were used for experiments; note that KO was not complete and depended on CRISPR efficiency within the pooled cells. However, overall, reductions in PRRC2B levels were consistent over multiple independent infections, and only pools that achieved at least an 80% reduction in both isoforms were used for further analysis. Experiments were performed on freshly prepared cells from independent infections, and maintained at low passage to avoid random genetic shifts within the population that occur with long passaging.

### Sequence alignment and motif analysis

PRRC2 protein sequences were identified in public sequence databases by blast searches starting with the human PRRC2B protein and then other identified vertebrate sequences. Sequences were multiply aligned using the GLAM2 [23] and blast programs, and multiple alignments were aligned to each other using the COMPASS program [24]. Multiple alignment sequence logos visualization is as previously described [25]. To quantify the occurrence of RG dipeptides in PRRC2B_L_, the product of the 164 occurrences of Arg and 200 occurrences of Gly was divided by the number of dipeptides in the PRRC2B_L_ protein (2228), giving a value of 14.72. This expected value is not significantly different from the observed value of 15 [26, 27]. To test for the overabundance of RG dipeptides in exon 16, a right sided Fisher’s exact test (FET) was made with the number of RG dipeptides in exon 16 (11), the number not in exon 16 (4), the number of dipeptides in exon 16 (694 aa long), and the number not in exon 16 (1533 aa long). Similar calculations were made for the RG-rich domain, using the corresponding values: 7 RG dipeptides in the domain, 8 RG dipeptides not in the domain, 50 dipeptides in the domain, and 2177 dipeptides not in the domain.

### Cloning of PRRC2B constructs

RNA from HEK 293T cells was purified using the Monarch total RNA miniprep kit (NEB). cDNA was reverse-transcribed with SuperScript III (Invitrogen). Full-length PRRC2B long and short isoforms were amplified from cDNA by PCR (KAPA HiFi HotStart Ready Mix PCR Kit) with primers containing restriction sites for KpnI and EcorV. The reverse primer also contained the HA tag sequence and two stop codons. The amplified products were then inserted into the pcDNA expression vector using the aforementioned restriction enzymes. PRRC2B ΔBAT2 was constructed using whole plasmid PCR with phosphorylated primers: Forward primer: 5′-ATGCTCCGCCCTCAGAATGTG-3′ Reverse primer: 5′-GGTACCAAGCTTGGGTCTCC-3′. pcDNA PRRC2B_L_/_S_ plasmids were used as templates. The reaction was then treated by DpnI for 2 h to remove the original plasmid. The PCR product was cleaned using the QIAquick PCR Purification Kit and ligated into pCDNA3. ΔRG PRRC2B construct was created in the same manner using the following primers: Forward: 5-‘ CTGCGAGAGTTTGCGCGGC-3′, Reverse: 5′-GACCCCAAAGGCTTGCTCC-3′.

### Polysome Profiling

HEK 293T cells were incubated with 100 μg/ml cycloheximide (CHX, Sigma) for 5 min and then washed twice with cold PBS buffer containing 100 μg/ml CHX. The cells were collected and lysed for 10 min while rotating at 4°C in lysis buffer (K-HEPES 25 mM Tris (pH 8), 200 mM KAc, 15 mM MgAc_2_ 1% NP-40, 0.5% DOC, with 100 μg/ml CHX, 1.0 mM DTT, 1.0 mM PMSF in DEPC-treated water). The lysed samples were centrifuged at 5000 × g at 4°C for 5 min. The cleared lysates were loaded onto a 10–50% sucrose gradient and centrifuged at 38 000 rpm in an SW41 rotor for 1h 45 min at 4°C. Gradients were fractionated into 33–34 fractions, and the optical density at 254 nm was continuously recorded using the ISCO absorbance detector UA-6. Every three consecutive fractions of collected samples were combined and concentrated using an Amicon Ultra-4 10kd Centrifugal Filter for western blotting.

### Co-immunoprecipitation

HEK 293T cells were transiently transfected for 24 h with HA-tagged PRRC2B constructs or HA-mCherry as a control. Cells were lysed in buffer B (20 mM HEPES-KOH [pH 7.6], 100 mM KCl, 0.5 mM EDTA, 0.4% NP-40, 20% glycerol) supplemented with protease and phosphatase inhibitors (Sigma), 0.1 mM phenylmethylsulfonyl fluoride (PMSF). 100 mg/ml RNase A (Thermo Scientific, EN0531) was added to relevant samples. Lysates were incubated with anti-HA beads (Sigma) for 2 h at 4°C. Following 3 washes with buffer B, the beads were incubated for 5 min with elution buffer (100mM Tris-HCl, 5% SDS, PH 7.4) and analyzed by mass spectrometry or western blotting on 8% Tris-Glycine gels.

### RIP assay

HEK 293T cells plated on 15 cm plates were transfected with HA-tagged PRRC2B constructs or HA-mCherry as a control, and after 5 h, incubated in EBSS for 48 h. Lysates were prepared and immunoprecipitated (IP) with anti-HA antibody beads as described above, except that washes were done in NT-2 buffer (50 mM Tris, pH 7.4, 150 mM NaCl, 1 mM MgCl_2_, 0.05% Nonidet *P*-40). 20% of beads were subjected to elution for purifying protein for western blotting, and the remainder was treated with Proteinase K (NEB P8107S) in NT-2 buffer while shaking at 55°C for 30 min. RNA was eluted from total cell lysate or the Proteinase K-treated IP using Tri-reagent (Sigma-Aldrich, cat# T9424). RNA was then subjected to oligo(dT) purification using Dynabeads™ mRNA DIRECT™ Purification Kit (Invitrogen, cat# 61 012) per manufacturer’s instructions. cDNA was prepared from equal quantities of purified mRNA from total cell lysates or eluted IPs and subjected to qRT-PCR as described below.

### Quantitative real-time PCR

NT or PRRC2B KO cells plated in 6-well dishes were grown in either DMEM, EBSS, or EBSS with 50 nM actinomycin D (Sigma-Aldrich, cat# A1410) for 24 h. Alternatively, for rescue experiments, KO cells were transfected with empty pcDNA, PRRC2B_L_-HA, or PRRC2B_S_-HA plasmids, and then, 5 h later, starved for 48 h in EBSS media. Cell lysates were prepared and RNA extracted using the Monarch total RNA miniprep kit according to the manufacturer’s instructions. The lysate was spiked with 10 ng Firefly luciferase mRNA (Promega, cat# L4561) for every 100 000 cells. 750 ng total RNA was transcribed into cDNA using AzuraQuant™ II cDNA Synthesis Kit (cat# AZ-2504). qRT-PCR was done with AzuraView™ GreenFast qPCR Blue Mix LR (cat# AZ-2305) on QuantStudio™ 5 Real-Time PCR System. Fold changes were calculated by the ΔΔCt method, normalizing to levels of luciferase mRNA. Primers used can be found in Supplementary Table S5. For rescue experiments, cells were transfected in parallel for the preparation of lysates for western blotting to confirm PRRC2B isoform levels.

### Nascent protein labeling and purification

*De novo* synthesized proteins were assayed as previously described [28]. HEK 293T PRRC2B KO and NT cells were grown in DMEM for basal conditions, or incubated in EBSS for 48 h (starvation), followed by 1 h recovery in DMEM Methionine-free media (Gibco, cat# 21 013 024) (recovery). For all conditions, cells were incubated in the appropriate methionine-free media (DMEM or EBSS) supplemented with 25 μM Click-iT AHA (Thermo Fisher Scientific, cat# C10102) for 1 h. Lysates were quantified using Pierce™ BCA Protein Assay (Thermo Scientific, cat# 23 227), and between 80 and 100 µg of proteins were labeled with biotin using the Click-iT protein reaction kit (Invitrogen, cat# C10276). Biotinylated proteins were precipitated according to the manufacturer’s instructions and kept at −20°C until further use. Proteins were resuspended, quantified again using BCA, and equal amounts were loaded on Pierce NeutrAvidin Agarose beads (Thermo Scientific™, cat# 29 200) for 2 h at 4°C. Purified biotinylated proteins were analyzed by western blotting for RPS protein levels. Band density was quantitated using Image Studio, and the density of RPS protein bands was normalized to calnexin, used as a non-TOP loading control.

### Western Blot

Cells were lysed with B buffer or RIPA (20 mM Tris, pH 8.5, 0.1% NP40, 150 mM NaCl, 0.5% sodium deoxycholate, 0.1% SDS) supplemented with 10 µl/ml 0.1 M PMSF (Sigma-Aldrich, cat# 93 482) and 1% protease inhibitor (Sigma-Aldrich, cat# P8340), and electrophoresis and western blots were performed according to standard protocols. Blots were stained with Ponceau S (Sigma, cat# P7170) prior to incubation with primary antibodies. Secondary antibodies were either horseradish peroxidase (HRP)-conjugated goat anti-mouse (Jackson ImmunoResearch Labs, cat# 115–035-003) or anti-rabbit (Jackson ImmunoResearch, cat# 111–165-144), detected by enhanced chemiluminescence using SuperSignal™ West Pico PLUS Chemiluminescent Substrate (Thermo Scientific, 34 580).

### Mass spectrometry

*Interactome analysis*. PRRC2B_L_-HA and PRRC2B_S_-HA and control HA-mCherry immuno-precipitates were subjected to in-solution tryptic digestion, or for methylation analysis, chymotrypsin digestion, and analyzed by LC/MS as previously described [29]. Samples were loaded using split-less nano-Ultra Performance Liquid Chromatography (10 kpsi nanoAcquity; Waters, Milford, MA, USA) coupled online through a nanoESI emitter (10 μm tip; New Objective; Woburn, MA, USA) to a quadrupole orbitrap mass spectrometer (Q Exactive Plus, Thermo Scientific) using a FlexIon nanospray apparatus (Proxeon). Data was acquired in data-dependent acquisition (DDA) mode, using a Top10 method. MS1 resolution was set to 70 000 (at 200 m/z), mass range of 375–1500 m/z, AGC of 1e6, and maximum injection time was set to 60 msec. MS2 resolution was set to 17 500, quadrupole isolation 1.7m/z, AGC of 1e5, NCE of 27%, dynamic exclusion of 25 s, and maximum injection time of 60 msec. Raw data were processed with MaxQuant v1.6.6.0 [30]. The data was searched with the Andromeda search engine against the human (*Homo sapiens*) protein databases as downloaded from Uniprot (http://www.uniprot.org), appended with common lab protein contaminants as previously described [29]. The quantitative comparisons were calculated using Perseus v1.6.0.7 [31]. Decoy hits were filtered out, and only proteins that were detected in at least two replicates of at least one experimental group were kept. Empty intensity values were imputed using the corresponding Perseus function. A Student’s *t*-test, after logarithmic transformation, was used to identify significant differences between the experimental groups across the biological replica. Fold changes were calculated based on the ratio of geometric means of the different experimental groups. Interactions were considered significant for proteins with at least two peptides, whose abundance was enriched in PRRC2B_L_ or PRRC2B_S_ immunoprecipitates over mCherry immunoprecipitate more than 2-fold, FDR < 0.05.

*Post-translational modification analysis*. Raw data were processed with Proteome Discoverer v2.4 informatics platform (Thermo Scientific) and searched against the human protein database as above. The search was done with the SequestHT and Andromeda 2.0 search engines. Search parameters were defined to include chymotryptic peptides with up to two missed cleavages allowed. Fixed modification was set to carbamidomethylation of cysteines. Variable modifications were set to oxidation of methionines, protein *N*-terminal acetylation, and arginine methylation. Peptide and protein identifications were filtered at an FDR of 1% using the Percolator software.

### Immunofluorescence staining

U2OS G3BP-GFP cells were plated in 6-well dishes for transfection with the indicated plasmids. 24 h post-transfection, the cells were trypsinized and replated on ibidi μ-Slide 8 Well plates (ibidi, cat# 80 806). The following day, the cells were treated with 100 µM sodium arsenite (NaAsO_2_) (Sigma, cat# 71 287) for 30 min. The cells were washed with PBS and fixed using 4% PFA (Electron Microscopy Science, 15 710). Permeabilization was performed with PBS + 0.1% Triton X-100 (PBT), followed by blocking in PBS containing 5% normal goat serum (NGS) (Biological Industries, cat# 04–009-1A) for 1 h. Wells were incubated with primary antibody, mouse anti-HA (Biolegend, cat# 901 501), diluted in blocking solution for 1 h at room temperature, followed by 1 h with Alexa Fluor® 555 goat anti-mouse IgG (Invitrogen, cat# A21424) and then stained with DAPI. The wells were kept in Fluoromount G (Southern Biotech, cat# 0100–10) and visualized with a Zeiss LSM900 confocal microscope, 40x C-Apochromat water-immersion objective, N.A. 1.2, with excitation and emission wavelengths of 555 nm and 565 nm for Alexa Fluor® 555, and 488 nm and 525 nm for visualization of GFP. Images were processed using ZEN software (version 2.4). For co-localization with mitochondria, HeLa cells grown on ibidi μ-Slide 8 Well plates were transfected with HA-PRRC2B_L_ and stained with MitoTracker™ Red CMXRos (Invitrogen, cat# M7512) for 30 min. Fixation, staining for HA, and imaging were performed as described above, using Alexa Fluor® 488 goat anti-mouse IgG (Invitrogen, cat# A11029), and excitation and emission wavelengths 488 and 525 nm, respectively.

### RNA deep sequencing

RNA-seq libraries were prepared at the Crown Genomics Institute of the Nancy and Stephen Grand Israel National Center for Personalized Medicine, Weizmann Institute of Science. Libraries were prepared using the TruSeq Stranded mRNA-seq kit (Illumina) according to the manufacturer's protocol. Briefly, the polyA fraction (mRNA) was purified from 500 ng of total input RNA from PRRC2B KO and control NT cells grown in DMEM or EBSS media for 48 h, followed by fragmentation and the generation of double-stranded cDNA. After Agencourt Ampure XP beads cleanup (Beckman Coulter), A base addition, adapter ligation, and PCR amplification steps were performed. Libraries were quantified by Qubit (Thermo Fisher Scientific) and TapeStation (Agilent). Sequencing was done on Novaseq Plus X 1.5B 300 cycles kit, allocating ∼50M reads per sample (Illumina; paired end sequencing).

Adapter trimming, alignment to hg38, and statistical analyses were performed with the UTAP pipeline [32], which utilizes STAR version 2.7.10a, cutadapt version 4.1 DESeq2(v1.36.0) for normalization and differential expression analysis. Importantly, batch was included as a co-factor in the statistical model. Additional DESeq2 parameters included: betaPrior = TRUE, cooksCutoff = FALSE, and independentFiltering = FALSE. The sva R package was used to apply batch correction to the log2-normalized counts, and the resulting output was used for PCA analysis. The data were analyzed first with all four samples, and then with only EBSS samples, as total RNA content was greatly reduced in these cells compared to DMSO. It should be noted that by loading equal amounts of total RNA for the RNA-seq protocol, the overall reduction in RNA abundance that occurs during starvation was masked. Thus, the standard normalization procedure distorted the comparison between DMEM and EBSS samples. As such, the genes whose levels actually **decreased** in EBSS samples, which were the majority of genes, were artificially scored as unchanged versus their levels in basal conditions, while those that truly remain unchanged, or decreased to a smaller extent than average, gave the illusion of increasing in abundance. Accordingly, the TOP genes, which were defined by the analysis as **increased** DEGs during starvation, were actually unchanged compared to basal conditions. Criteria for passing the differential expression test were: BaseMean > 10, │log2FC│ > 0.263 (FC > 1.2), and *P* < 0.05.

### Statistical analysis

Statistical analysis of large datasets (MS, RNA-seq) and sequence analysis is provided in the respective sections of the Materials and Methods above. Additional analysis was done using GraphPad Prism version 10.4 by unpaired 2-tailed T-tests, using Welch’s correction when variance was unequal, or 1-sample 2-tailed T-tests when compared to control samples with no SD, 1-way ANOVA with Tukey’s post-hoc comparison test, two-way ANOVA with Sidak’s post-hoc multi-comparison analysis, or two-sided Fisher’s Exact Test contingency analysis, as indicated in figures, with *P* < 0.05 considered statistically significant. Statistical significance of overlap for intersecting sets was determined using the http://nemates.org/MA/progs/overlap_stats.html server.

### Websites/Database Referencing

The following programs, servers and databases were used for data analysis and visualization: sequence analysis and alignment: Uniprot (http://www.uniprot.org); UCSC genome browser (https://genome.ucsc.edu/cgi-bin/hgGateway); GALM2 (https://meme-suite.org/meme/tools/glam2) [23]; COMPASS (http://prodata.swmed.edu/compass/compass.php) [24]; protein structure and motif predictions: Alphafold-3 (https://golgi.sandbox.google.com/, queries Q5JSZ5, Q5JSZ5-5) [33], RosettaFold (https://github.com/RosettaCommons/RoseTTAFold) [34], and DisoRDPbind (http://biomine.cs.vcu.edu/servers/DisoRDPbind/) [35]; Gene annotation and pathway analysis: GeneAnalytics (https://ga.genecards.org/#input) [36], WEB-based GEne SeT AnaLysis Toolkit (https://www.webgestalt.org) [37], STRING (https://string-db.org/) [38]; Data visualization: Volcano plots (https://goedhart.shinyapps.io/VolcaNoseR/); Venn Diagrams and statistics of overlap: (https://bioinformatics.psb.ugent.be/webtools/Venn/, https://www.meta-chart.com/venn#/display), and (http://nemates.org/MA/progs/overlap_stats.html).

## Results

### PRRC2B has two splice isoforms, PRRC2B_L_ and PRRC2B_S_

**Figure 1.**
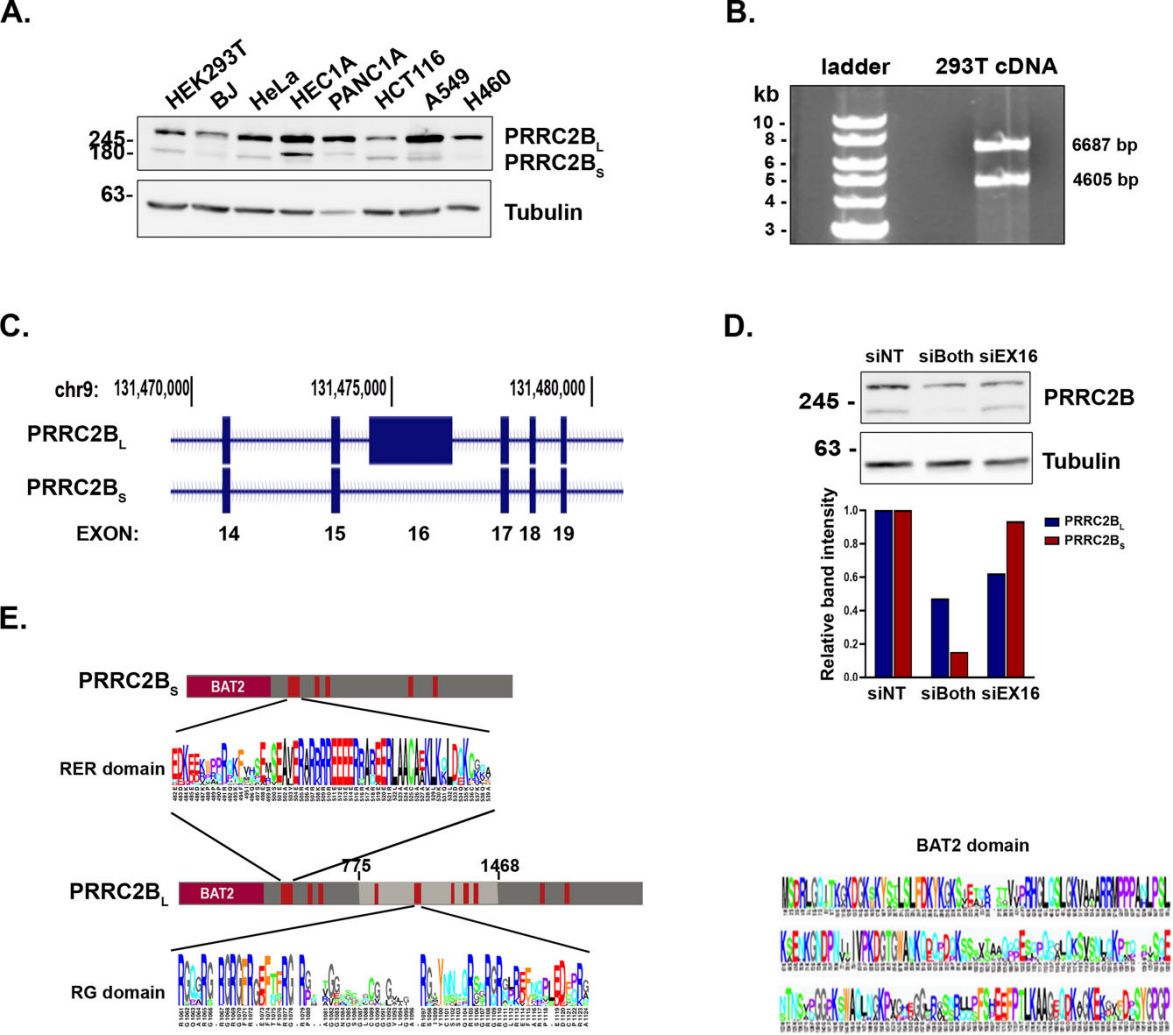
PRRC2B is expressed as long and short isoforms in human cell lines. (**A**) Western blots of the indicated cell lines for PRRC2B. Tubulin was used as a loading control. (**B**) Products of PCR performed on cDNA isolated from HEK 293T cells using primers from 5′ and 3′ UTRs. (**C**) Portion of the PRRC2B genomic locus as represented on the UCSC genome browser depicting two splice isoforms, zooming in on exon 16 and surrounding exons. Numbers on top represent chromosomal position. (**D**) HEK 293T cells were transfected with control non-targeting (NT) siRNA, or siRNA to regions common to both isoforms (siBoth), or only exon 16 (siEx16), and after 3 days, lysates were subjected to a western blot for PRRC2B and tubulin as a loading control. Band intensities of PRRC2B isoforms under each KD condition were quantitated by densitometry and normalized to respective levels of tubulin. Graph shows levels of PRRC2B_L_ and PRRCB_S_ relative to levels of each in siNT KD, levels of each isoform in siNT were set at 1. (**E**) Schematics of PRRC2B_L_ and PRRC2B_S_ protein structures, with regions of high conservation colored red. Region derived from exon 16 in PRRC2B_L_ is colored light grey. Multiple sequence alignment within the indicated BAT2, RER, and RG-rich domains is shown according to the human amino acid position numbers.

Western blotting of various human cell lines for PRRC2B expression with an antibody that recognizes the C-terminus of the protein (amino acids 2102–2139) revealed two bands of different sizes: a slower migrating band running above the 245 kDa marker that presumably corresponds to the previously reported protein [18,20], and a faster migrating, shorter form running just above the 180 kDa marker (Fig. 1A, Supplementary Fig. S1A). We observed these two forms in all cell lines assayed, including fibroblasts and cancer cells of different origins, at different proportions, with the long form predominating. The expression of these isoforms was also assessed upon exposure of HEK 293T cells to various stress conditions, including: ER stress (thapsigargin treatment), arsenite treatment, chemical hypoxia (CoCl_2_), and nutrient starvation (EBSS) (Supplementary Fig. S1B). An increase in the ratio between the short isoform and the long one was detected in response to ER stress and hypoxia, and conversely, the ratio was decreased during nutrient starvation. To investigate the origin of these forms, we performed PCR on cDNA generated from HEK293T cells using primers from either end of the predicted coding sequence of PRRC2B. Two PCR products were detected, the longer of which matched the predicted size of the 6687 bp long cDNA encoding the reported PRRC2B protein of 2229 amino acids (Fig. 1B). The smaller band was sequenced to determine its identity, specifying a 4605 bp long cDNA encoding a protein identical to the reported PRRC2B, but lacking nucleotides 2325–4407 (amino acids 775–1468). We noted multiple alternative splicing events within the PRRC2B gene upon analysis of NCBI RefSeq gene products on the UCSC genome browser (https://genome.ucsc.edu/cgi-bin/hgGateway, human assembly GRCh38/hg38). Specifically, the smaller form sequenced in HEK293T cells corresponds to a splice variant lacking a single long exon (Exon 16) (Fig. 1C). Importantly, levels of both protein bands were reduced upon transfection of HEK 293T cells with a pool of siRNA targeting regions common to both the long and short isoforms within the CDS and 3′ UTR (siBOTH), but only the longer protein showed reduced expression upon transfection of siRNA targeting the CDS within exon 16 (siEx16) (Fig. 1D). This confirms the identity of the protein isoforms, which we will hereafter refer to as PRRC2B-long (PRRC2B_L_) and PRRC2B-short (PRRC2B_S_), respectively.

By this analysis, both PRRC2B_L_ and PRRC2B_S_ share the same *N*- and *C*-termini, the former consisting of a 200 amino acid conserved region termed the BAT2 domain (Fig. 1E) [39]. However, the short isoform lacks the central part of the protein, including a conserved region encompassing aa 1061–1124, which contains 7 di-repeats of the amino acid Arg followed by Gly. Notably, this region, which represents less than 3% of the total protein length, contains nearly half of the total 15 RG dipeptides found in the entire protein; 11 RG dipeptides are found within the entire exon 16. The enrichment of RG dipeptides in both exon 16 and the conserved region of aa 1061–1124 is statistically significant (right-sided Fisher’s exact test (FET), *p *= 0.001 and *p *= 1.13e–07, respectively), as determined by calculations detailed in [26, 27]. We thus refer to the conserved region hereafter as the RG-rich domain. Moreover, multiple alignments of this region across vertebrate families (Fig. 1E, bottom) showed that the RG-rich region is highly conserved (Mann–Whitney U test for whether the sites outside exon 16 are less conserved than those in it, after weight-correction for the occurrence of RG sites in human proteins; *P *= 0.037). Considering that conserved RGG/RG motifs are found in many RNA-binding proteins, and have been shown to mediate interactions with both nucleotides and proteins [40], the significant enrichment and conservation of the RG-rich domain imply functional importance of this region.

### PRRC2B_L_ and PRRC2B_S_ share common structural and functional features

We used the Alphafold-3 (https://golgi.sandbox.google.com/, queries Q5JSZ5, Q5JSZ5-5) or RoseTTAfold servers [33, 34] to model the structures of both long and short PRRC2B isoforms (Supplementary Fig. S1C). PRRC2B_L_ is predicted to be a mostly unstructured protein with almost no internal contacts or folded domains. As such, the predictions are scored as mostly weakly reliable or unreliable, with the exception of a long central helix that scored mostly as highly reliable (see legend of Supplementary Fig. S1C for details). This prominent helix (residues 492–550) contains a highly charged surface (Supplementary Fig. S1C, bottom), bearing the conserved sequence RKRREEEERR (see Fig. 1E). This sequence (referred hereafter as the RER motif) is conserved in PRRC2A and PRRC2C as well (Supplementary Fig. S1D). Analysis of the sequence of PRRC2B by the prediction algorithm DisoRDPbind (http://biomine.cs.vcu.edu/servers/DisoRDPbind/) [35] showed several peaks with predicted RNA binding propensity, the strongest of which corresponded to this region (Supplementary Fig. S1E). This helix is surrounded by shorter helices and unstructured coils that differ in length and position in the five models predicted by Alphafold or RoseTTAfold. None of them contacts the long helix, hence a folded domain is not formed. PRRC2B_s_ is similarly unstructured, with the long, charged α-helix prominent in its center, although the BAT2 domain and the other smaller helices are differentially positioned compared to PRRC2B_L_ (Supplementary Fig. S1C). These predictions are consistent with previous descriptions of PRRC2 family members as intrinsically disordered proteins [19], a feature common to RNA-binding proteins and proteins that form membrane-less organelles [41].

**Figure 2.**
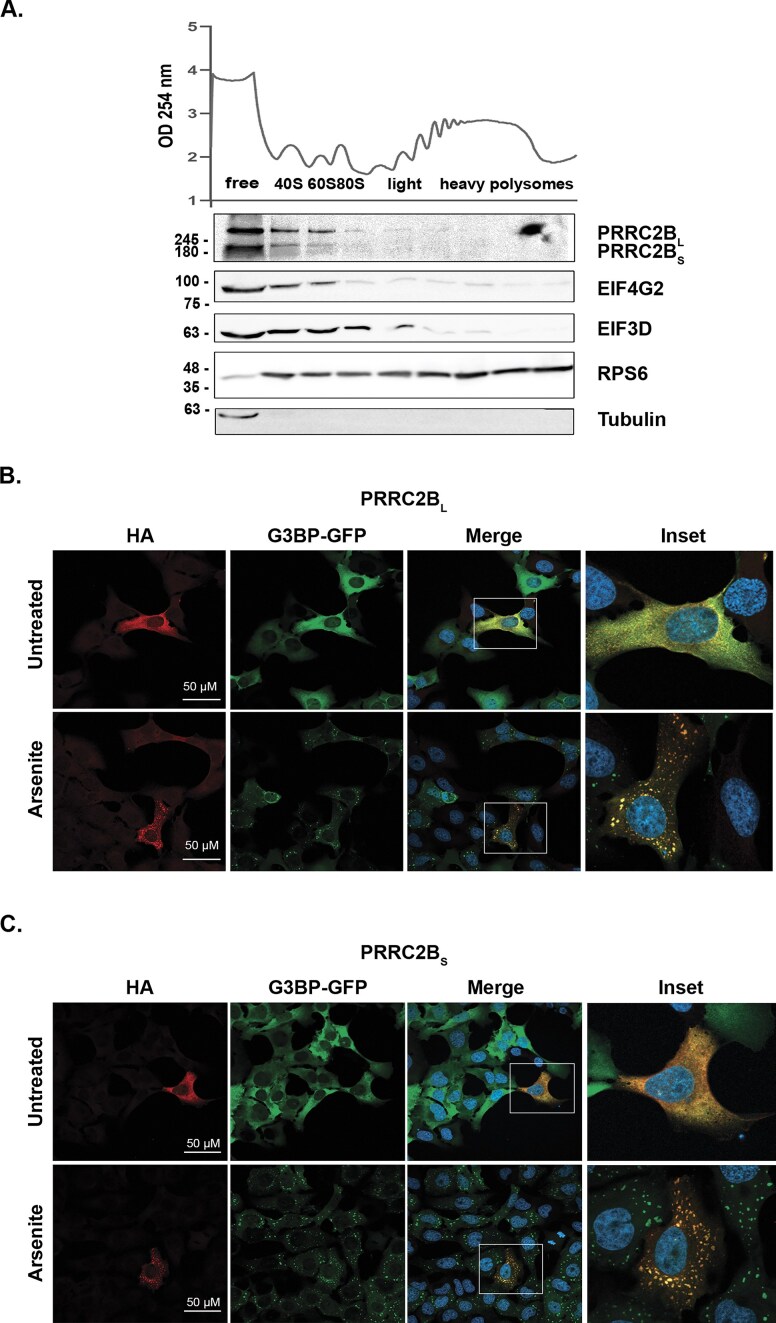
Both PRRC2B isoforms exhibit features of translation initiation factors. (**A**) Polysome profiling of HEK 293T cell lysate. Lysates were fractionated on 10–50% sucrose gradients, and every three pooled fractions were subjected to western blotting for the indicated proteins. The graph on top shows the presence of RNA (OD_254_) along the fractions, with corresponding positions of ribosomes indicated. Shown is representative data from one of two independent experiments. (**B** and **C**) U2OS cells stably expressing GFP-G3BP were transfected with HA-tagged PRRC2B_L_ (**E**) or PRRC2B_S_ (**F**) and treated with 100 µM sodium arsenite or left untreated for 30 min. Cells were fixed and stained with anti-HA and DAPI to detect nuclei. Insets at right show enlargements of corresponding boxed areas. Shown are representatives of 8–10 fields examined.

Previous reports have indicated that PRRC2B functions as a translation initiation factor and that it co-migrates with the 40S ribosome, and also with polysomes, to varying degrees, upon polysome fractionation [18–20]. To explore whether the loss of exon 16 in PRRC2B_S_ confers functional differences to the isoform in this regard, we purified polysomes from HEK 293T cell lysates endogenously expressing both long and short PRRC2B by fractionation on 10–50% sucrose gradients. Our assay validated previous results for PRRC2B_L_, which was mainly detected in association with free ribosomal subunits, particularly the 40S fractions, similar to other canonical and non-canonical translation initiation factors, such as EIF3D and EIF4G2, respectively (Fig. 2A). Notably, the short isoform showed an identical fractionation pattern to the long isoform, in proportion to its expression levels. The long isoform was also faintly observed in fractions corresponding to the polysomes.

PRRC2 family members were previously shown to be components of stress granules [17], membraneless organelles acting as reservoirs of stalled translation initiation complexes, including 40S ribosome, translation initiation factors, mRNAs, and RBPs, that accumulate during cell stress. To determine whether both isoforms of PRRC2B shift to stress granules in response to sodium arsenite treatment, we cloned long and short isoforms individually into pcDNA expression plasmids, fused to an HA tag at the *C*-termini, and transfected them into U2OS cells expressing GFP-tagged G3BP, a stress granule component. Cells were then treated with sodium arsenite to induce stress. Staining for HA indicated that both PRRC2B isoforms shifted from a cytoplasmic diffuse localization under basal conditions to GFP-marked, punctate stress granules upon treatment of cells with sodium arsenite (Fig. 2B and C). Similar results were observed with a variant of PRRC2B_L_ in which the RG-rich domain was deleted (Supplementary Fig. S1F). Thus, loss of the central region containing the RG domain does not impair PRRC2B_S_’s ability to globally bind initiation complexes and to translocate to stress granules.

### The long and short PRRC2B isoforms have different protein interactomes

**Figure 3.**
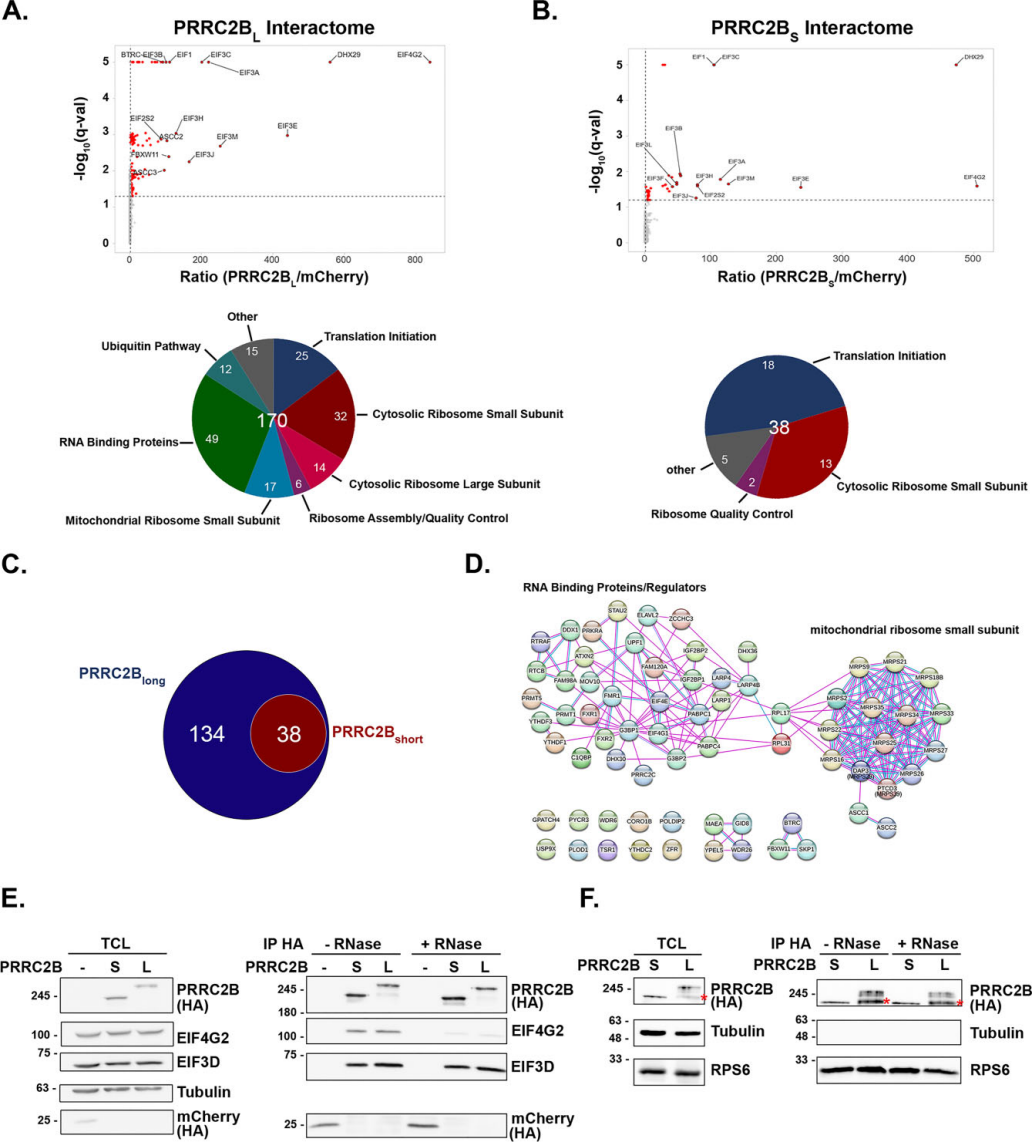
PRRC2B_L_ and PRRC2B_S_ have different protein interactomes. (**A-D**) HEK 293T cells were transiently transfected with pcDNA plasmids expressing HA-mCherry as a control, HA-tagged PRRC2B_L_ or PRRC2B_S_. Twenty-four hours after transfection, HA–protein complexes were co-immunoprecipitated and analyzed by Mass Spec. (**A, B**) Volcano plot of the fold-ratio of the abundance of the detected proteins in PRRC2B_L_ (**A**) or PRRC2B_S_ (**B**) versus control IP samples, versus their significance expressed as -log_10_  *P*-value. Top proteins with significantly increased abundance are indicated in red for each isoform, highlighting translation initiation factors. Pie charts beneath graphs indicate the distribution of interacting proteins within the indicated categories. (**C**) Venn diagram depicting overlap between PRRC2B_L_ and PRRC2B_S_ interactomes. (**D**) String network analysis of a set of 69 proteins that show significant enrichment (FC > 2, *p *< 0.05) in PRRC2B_L_ interactome compared to PRRC2B_S_ interactome. (**E, F**) HEK 293T cells transiently transfected with pcDNA plasmids expressing HA-mCherry as a control, HA-tagged PRRC2B_L_ or PRRC2B_S_ were immunoprecipitated with anti-HA antibodies in the absence or presence of 100mg/ml RNase A, and lysates and IPs were subjected to western blot analysis for the indicated proteins. Tubulin was used as a loading control. Red asterisks in (**F**) denote a degradation product of PRRC2B_L_, which increases during the IP procedure. Shown are representative blots of 4 (**E**) or 2 (**F**) independent experiments.

To further investigate the functional differences between the two isoforms, we examined the protein interactome of each isoform. To this end, we transfected HEK 293T cells with C-terminally HA-tagged PRRC2B_L_ or PRRC2B_S_ and immunoprecipitated the tagged isoforms and their interacting proteins with anti-HA agarose beads. Eluates were analyzed by LC-mass spectrometry (IP-MS). The tagged proteins were expressed at similar levels to allow a reliable comparison of their pulled-down interactomes, and to avoid artifactual interactions stemming from significant over-expression, their levels were minimized to near endogenous expression levels (Supplementary Fig. S2A). A quantitative comparison was performed between identified proteins in the IP eluates of the two PRRC2B-HA isoforms and the HA-mCherry control, and between one another. In total, 170 specific candidate interacting proteins (≥2 unique peptides, FDR < 0.05, and > 2-fold enrichment over control HA-cherry IP) were identified for the long isoform, and a smaller set of 38 proteins interacted with the short isoform, all of which also interacted with the long isoform (Supplementary Table S1, Fig. 3A–C). The larger number of interactors for PRRC2B_L_ is presumably due to the necessity of the large middle portion of the protein derived from exon 16 for these interactions. The top interactor of both isoforms was the non-canonical translation initiation factor EIF4G2, followed by DHX29, an RNA helicase involved in translation initiation, and translation initiation factors EIF1 and (subunits of) EIF3 (Supplementary Table S1, Fig. 3A and B). Many additional initiation factors showed a significant yet lower abundance in the respective IPs (Supplementary Table S1, Fig. 3A and B). Notably, both isoforms bound cytosolic ribosomal proteins, mostly of the small ribosomal subunit, consistent with their co-fractionation with 40S ribosomes observed above (Fig. 2C). Interestingly, even among the common interactors, the abundance of the initiation factors in the PRRC2B_L_ IP was roughly twice that of PRRC2B_S_ IP (Supplementary Table S1). Examining those proteins that preferentially interacted with PRRC2B_L_ but not PRRC2B_S_, by focusing on those that were enriched in abundance more than 2-fold in the former IP (*P *< 0.05), yielded a more focused set of 69 proteins. These mostly clustered into two main groups of proteins, as seen by network String analysis (Fig. 3D) [38]. One group consisted of RBPs, including m6a readers, RNA helicases, proteins mediating stress granule formation, and RBPs involved in selective mRNA stabilization associated with translation regulation, such as the PABP family members PABPC1 and PABPC4, LARP1, LARP4, and LARP4B. The Arg methyltransferases, PRMT1 and PRMT5, were also identified in the list of proteins that preferentially bind to PRRC2B_L_. The second group consisted of 14 proteins of the mitochondrial ribosomal small subunit (MRPS). This is quite surprising, as PRRC2B_L_ localizes to the cytosol with no signs of intramitochondrial staining (Supplementary Fig. S2B), suggesting interactions with MRPS occur in a cytosolic compartment prior to their mitochondrial translocation. Pathway analysis of the set of 69 proteins that preferentially interact with PRRC2B_L_ by GeneAnalytics GO Biological Processes confirmed these functional classifications (Supplementary Fig. S2C).

We validated the interactions of EIF3D and EIF4G2, representative of translation initiation factors, and a 40S ribosomal protein, RPS6, with both long and short PRRC2B isoforms by co-IP/western blots. Both isoforms co-IPed with EIF4G2, EIF3D, and RPS6 (Fig. 3E, F). The interactions with the translation initiation factors required the common NH_2_-terminal BAT2 domain (Supplementary Fig. S2D). Interestingly, immunoprecipitation in the presence of RNase A reduced the interaction with EIF4G2, but not with EIF3D or RPS6 (Fig. 3E and F), indicating that while the former requires RNA for binding, PRRC2B_L_ and PRRC2B_S_ can both recruit canonical translation factors and the 40S ribosome independently of mRNA.

Altogether, the differences in interactomes suggest that the short PRRC2B isoform has more limited functional capability compared to the long isoform, resulting from the removal of exon 16.

### PRRC2B_L_ specifically interacts with LARP1 and PABP proteins and binds to TOP mRNAs

**Figure 4.**
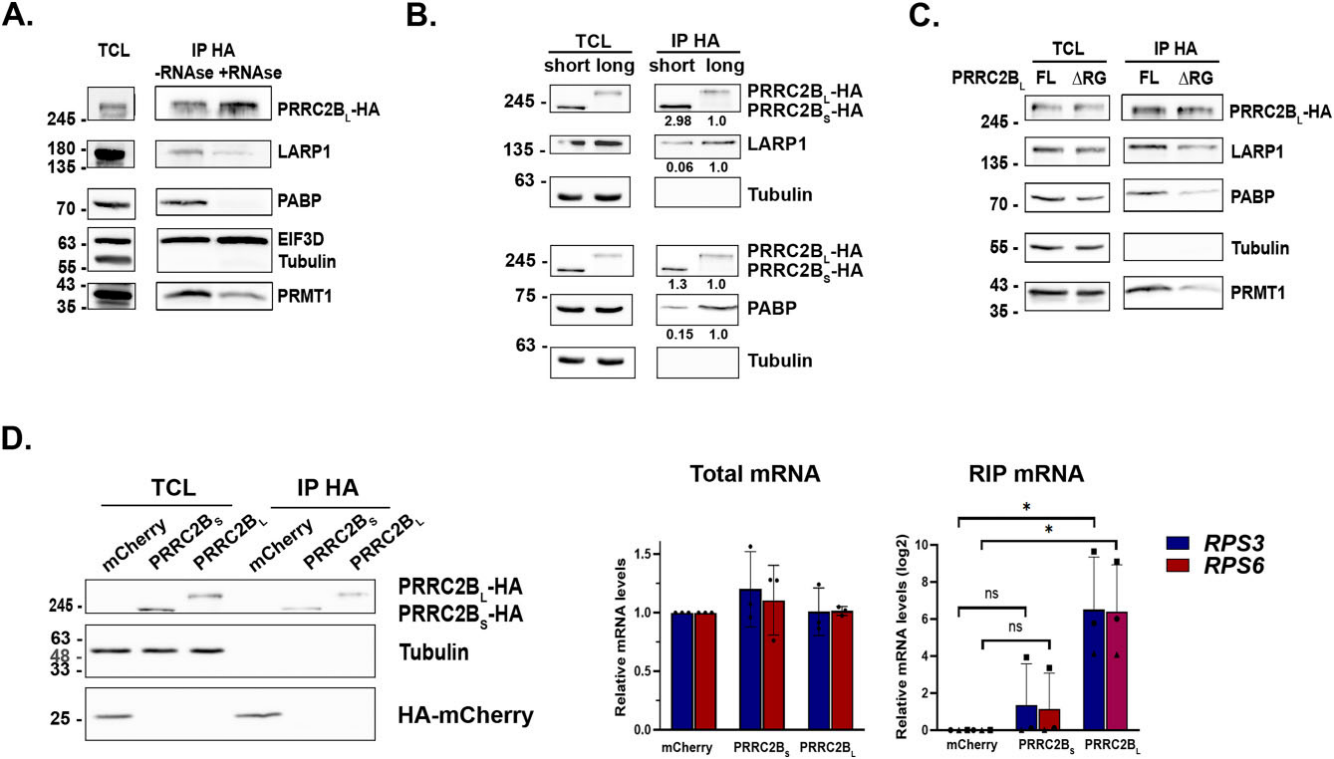
PRRC2B_L_-specific interactions with RBPs and TOP mRNAs. (**A**) HEK 293T cells transiently transfected with pcDNA plasmid expressing HA-tagged PRRC2B_L_ were immunoprecipitated with anti-HA antibodies in the absence or presence of 100 μg/ml RNase A, and lysates and IPs subjected to western blot analysis for the indicated proteins. Tubulin was used as a loading control. Shown is a representative blot of 3 experiments. (**B,C**) HEK 293T cells transiently transfected with pcDNA plasmids expressing HA-tagged PRRC2B_L_ or PRRC2B_S_ (**B**) or HA-tagged PRRC2B_L_ full length or ∆RG mutant (**C**) were immunoprecipitated with anti-HA antibodies, and lysates and IPs subjected to western blot analysis for the indicated proteins. Tubulin was used as a loading control. Shown are representative blots of 3 experiments. Numbers under blots in (**B**) represent relative protein levels as determined by densitometry, normalized to levels in PRRC2B_L_ IP, which was set as 1. The amounts of LARP1 and PABP in each IP were also normalized to levels of each PRRC2B isoform, to account for the greater levels of PRRC2B_S_ IPed compared to PRRC2B_L_. (**D**) RIP assay. HEK293T cells were transfected with either HA-mCherry, PRRC2B_L_-HA, or PRRC2B_S_-HA, starved for 48 h in EBSS media, and lysates were immunoprecipitated with anti-HA antibody-bound beads. Western blot with anti-HA antibodies shows levels of protein expressed in total cell lysate (TCL) and in HA IP in one representative experiment. Tubulin was used as a loading control. qRT-PCR for *RPS3* and *RPS6* was performed on total mRNA and mRNA that co-IPed with HA-tagged proteins (RIP mRNA). Graphs show relative mRNA levels, expressed as mean ± SD with individual data points of *n* = 3 independent biological experiments. For analysis of total lysate, fold-changes in mRNA levels were calculated by the ΔΔCt method and normalized to levels in mCherry, which was set as 1. One-way ANOVA with Tukey’s post-hoc multiple comparison test indicated no significant differences among the samples. For analysis of RIP, mRNA fold-changes were calculated by comparing to levels in the mCherry IP (ΔCt), and then corrected for any differences in protein levels of each isoform present in the IP. The graph shows log2-transformed relative fold-change compared to mCherry IP, with different-shaped datapoints corresponding to the individual experiments. Statistical significance was determined by 1-sample single-tailed *t*-tests with a hypothetical mean of 0, * *P *< 0.05. ns, not significant. Single-tailed tests were used since decreases below the baseline observed for control mCherry RIP are neither possible nor biologically meaningful.

We then focused on several of the exclusive PRRC2B_L_-interacting proteins in order to explore isoform-specific functions. First, the interactions with LARP1, PABP, and PRMT1 were validated by co-immunoprecipitation experiments with HA-tagged PRRC2B. All three proteins co-immunoprecipitated with PRRC2B_L_, and in contrast to EIF3D, the interactions were decreased upon RNase A treatment (Fig. 4A). Notably, and in agreement with the MS data, the co-IP between PRRC2B_S_ and both LARP1 and PABP was greatly reduced compared to that of PRRC2B_L_, despite the fact that the amount of short isoform IPed was greater (Fig. 4B). Deletion of the RG-rich domain, the putative RNA-binding region, from PRRC2B_L_ also reduced its binding to LARP1, PABP, and PRMT1 (Fig. 4C). The latter is particularly interesting as R1077 and R1079 within PRRC2B_L_’s RG-rich domain were identified as methylated by MS (Supplementary Fig. S3A, Supplementary Table S2). As these are predicted substrates for methylation by PRMT1, the main mammalian type I Arg methyltransferase [42], it is notable that this region is both required for interaction with PRMT1 and likely modified by the methylase.

LARP1 and PABP are RBPs that are involved in the regulation of 5′ TOP mRNA stability and translation. 5′ TOP mRNAs, which include most cytosolic ribosomal proteins and several translation factors, bear a characteristic 5′ terminal oligopyrimidine (TOP) motif that imparts special sensitivity to mTORC1 regulation, enabling efficient and immediate repression of translation initiation upon mTOR inhibition or inactivation [43, 44]. LARP1 binds both the 5′ TOP motif and the polyA tail [45–51] and thus specifically preserves TOP mRNA levels by promoting TOP mRNA stability [52–54]. Moreover, it facilitates post-transcription polyA elongation during amino acid starvation, and as a result preserves pools of TOP mRNA for immediate ribosome loading when metabolism is restored [28]. Both LARP1 and PRRC2B can bind components of the 40S ribosome in constitutive, basal conditions (Fig. 3 above and (([55], [56]), and the LARP1-40S ribosome complex was shown to stabilize TOP mRNAs following mTOR inhibition [53, 54]. Interestingly, PRRC2B can specifically bind CU rich sequences within the 5′ UTR of its interacting mRNAs [20], similar to LARP1 [57]. Considering these common features and the specific interactions between PRRC2B_L_ and LARP1 and PABP, we hypothesized that PRRC2B_L_ may be an additional RBP that participates in LARP1-mediated TOP mRNA regulation. Consistent with this, Gene Ontology (GO) annotation of the list of PRRC2B_L_ interacting proteins for enriched biological processes included the term “regulation of mRNA stability” (Supplementary Fig. S2C).

To determine whether, analogous to LARP1, PRRC2B binds TOP mRNAs, we performed an RNA-IP (RIP) assay following prolonged nutrient deprivation when mTOR is inhibited. Either HA-tagged PRRC2B_L_ or PRRC2B_S_, or mCherry as a control, was expressed in HEK 293T cells that were starved of amino acids and serum for 48 h (EBSS media). HA-tagged proteins were then immunoprecipitated from cell lysates, and associated mRNAs were quantified by qRT-PCR. *RPS3* and *RPS6* were used as representative TOP genes. Significantly, both *RP* mRNAs were enriched specifically within the PRRC2B_L_-HA immunoprecipitate, compared with the mCherry control. In contrast, levels of the RP mRNAs within the PRRC2B_S_-HA IP were not significantly different from those of the negative control (Fig. 4D). Moreover, they were significantly lower compared to those enriched in the PRRC2B_L_ IP (Supplementary Fig. S3B), indicating that *RPS3* and *RPS6* mRNAs interact exclusively with the long isoform. Thus, PRRC2B_L_, but not PRRC2B_S_, interacts with both LARP1 and TOP mRNAs. This is consistent with re-analysis of previously reported PAR-CLIP data performed in basal growth conditions [20] that identified 919 PRRC2B RNA-binding peaks that mapped to 762 unique protein-coding RNAs. By comparing this list to previously reported TOP mRNAs [43, 44], we identified 34 TOP mRNAs, including 26 ribosomal protein mRNAs and five translation factors, which contained PRRC2B binding sites. Interestingly, half of these TOP mRNAs retained binding to a fragment of PRRC2B referred to as P2 (aa 750–1500) [20], which almost conforms with PRRC2B_L_’s exon 16 (aa 775–1468). This, combined with our data, stresses the significance of the RG-rich domain that is exclusive to PRRC2B_L_ for interacting with TOP mRNAs, both under basal and starvation conditions.

### PRRC2B_L_ is necessary for maintaining TOP mRNA levels during amino acid starvation

**Figure 5.**
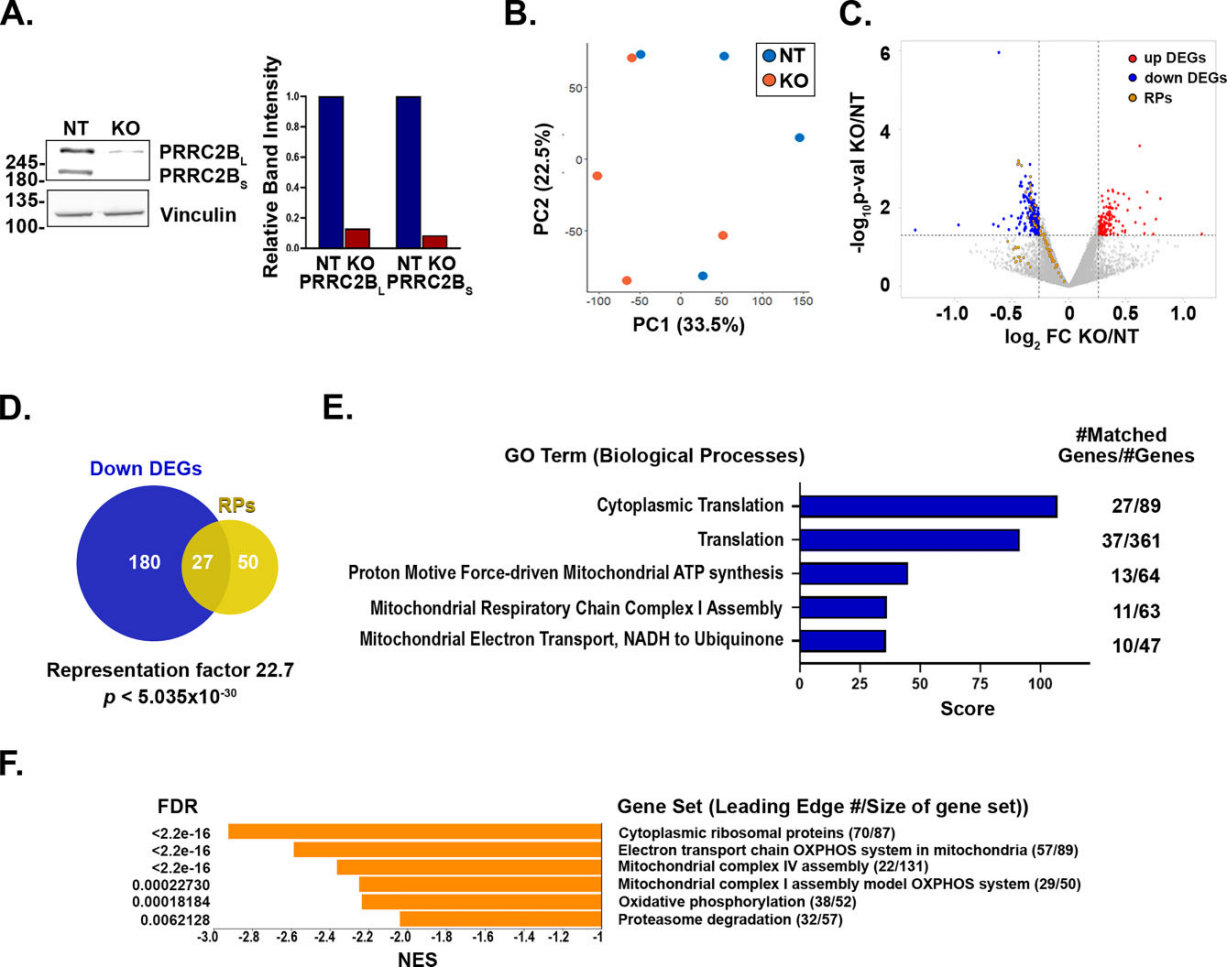
PRRC2B is necessary for the maintenance of TOP mRNA expression during starvation. (**A**) HEK 293T PRRC2B KO and control NT KO cells were generated by CRISPR-Cas9. Western blotting of pooled polyclonal KOs confirms a reduction in averaged PRRC2B expression. Vinculin was used as a loading control. The graph at right shows relative expression levels of PRRCB_L_ and PRRC2B_S_ in NT and KO cells, with NT levels set at 1, after normalization to vinculin levels in each lane. (**B-F**) RNA-seq was performed on PRRC2B KO and NT cells in basal conditions and following 48 h starvation in EBSS media. (**B**) PCA of RNA seq data from starved NT or PRRC2B KO cells. (**C**) Volcano plot of the fold-change in mRNA expression (protein-coding only) levels in PRRC2B KO versus NT control under starvation conditions, versus their significance expressed as -log_10_  *p*-value. Increased and decreased differentially expressed genes (DEGs) are indicated in red and blue, respectively. Yellow dots represent mRNAs encoding ribosomal proteins. (**D**) Venn diagram showing overlap between TOP mRNAs encoding ribosomal proteins (RPs) and the group of statistically significant down-regulated genes (down DEGs). (**E**) The set of 211 significantly decreased DEGs in starved PRRC2B KO cells versus control NT cells was analyzed by GeneAnalytics. The top five significant GO terms (Biological Processes) and scores are presented. The number of matched genes within the set out of the total number of genes in the particular biological process is indicated to the right of the graph. (**F**) The entire RNA-seq dataset was analyzed by WEB-based GEne SeT AnaLysis Toolkit (WebGestalt) gene set enrichment analysis (GSEA, https://www.webgestalt.org). Shown are the significant gene sets identified within the dataset, all of which have negative normalized enrichment scores (NES). The number of genes within the dataset (leading edge) out of the total number of genes in the particular gene set is indicated to the right of the graph.

To determine whether PRRC2B_L_’s binding to both LARP1 and TOP mRNAs has functional significance in regulating TOP mRNA, we used a pooled population of CRISPR-edited cells to perturb both isoforms of PRRC2B in HEK293T cells, using a non-targeting guide to generate control cells (NT) (Fig. 5A). Western blotting indicated an overall reduction in protein levels of at least 80%. Despite the heterogeneous, incomplete nature of the knock-out (KO) population, we used the pooled cells to minimize artifacts due to clonal selection and clone-specific off-target effects. We then performed RNA-seq on NT and PRRC2B KO HEK293T cells in basal conditions and following 48 h prolonged amino acid and serum starvation in EBSS media, focusing the analysis specifically on ribosomal proteins and other TOP mRNAs (Supplementary Tables S3, S4). Principal Component Analysis (PCA) indicated reproducibility of the replicates, which segregated mainly according to growth conditions (Supplementary Fig. S4A). First, we confirmed, as previously reported [52–54], that despite the global decrease in mRNA levels following starvation, levels of TOP mRNAs were maintained relative to the majority of mRNAs in control cells (Supplementary Fig. S4B, Supplementary Table S3). The dataset contained 111 mRNAs previously classified as TOP mRNAs [43, 44]. 90 of these (81%, *p *< 0.0001 by FET contingency analysis) were indeed observed within the group of DEGs whose levels were greater than the average majority of mRNAs (Supplementary Fig. S4B, Supplementary Table S3). Focusing only on the genes encoding ribosomal proteins (RPs), 75 out of 77 (97%) that were detected in the dataset exhibited statistically significant relative increased mRNA (*p *< 0.0001 by FET contingency analysis). Thus, the RNA-seq analysis confirms that TOP mRNA transcript levels are maintained during amino acid starvation in our cells. This process has previously been shown to involve LARP1-dependent protection and stabilization of the mRNAs [28, 46, 52–54].

To determine whether PRRC2B contributes to this regulation, we compared mRNA levels in NT and PRRC2B KO starved (EBSS) cells. Globally, there was a small degree of change in mRNA levels, as supported by PCA assessment of the dataset, which showed no differences between the clustering of KO and NT replicates (Fig. 5B). A group of 211 genes, excluding PRRC2B, showed a significant decrease in expression of at least 20% (Fig. 5C, Supplementary Table S4). Among these were 31 5′ TOP mRNAs, and 15 more non-pure TOP mRNAs that were documented as co-purifying with LARP1-40S ribosome complexes upon inhibition of mTOR [53]. Specifically, an examination of the 77 TOP genes encoding ribosomal proteins that were detected in the dataset indicated that all were reduced to some degree, 35% (27/77) significantly (Fig. 5C). Venn analysis indicated that this represents a significant enrichment within the set of mRNAs with decreased abundance (Fig. 5D). The effect was somewhat smaller when examining all TOP mRNAs; out of the 111 reported TOP mRNAs actually detected, 31 (28%) showed significantly reduced abundance (Supplementary Fig. S4C). Again, this was a significant enrichment (Supplementary Fig. S4D). GeneAnalytics GO analysis of the group of significantly down-regulated mRNAs yielded 18 categories with similar gene components that were reiterations on either translation or mitochondrial oxidative respiration/ATP generation (top five are shown in Fig. 5E). GSEA analysis of the dataset, which assesses even small changes that become significant when occurring within multiple components of the same pathway, also highlighted the ribosomal proteins as significantly downregulated (Fig. 5F, Supplementary Fig. S4E). Thus, the RNA-seq analysis indicates that the mechanism that maintains the expression of TOP mRNAs during starvation is impaired upon KO of PRRC2B.

### PRRC2B_L_ maintains TOP mRNA levels in a post-transcriptional manner during prolonged starvation

**Figure 6.**
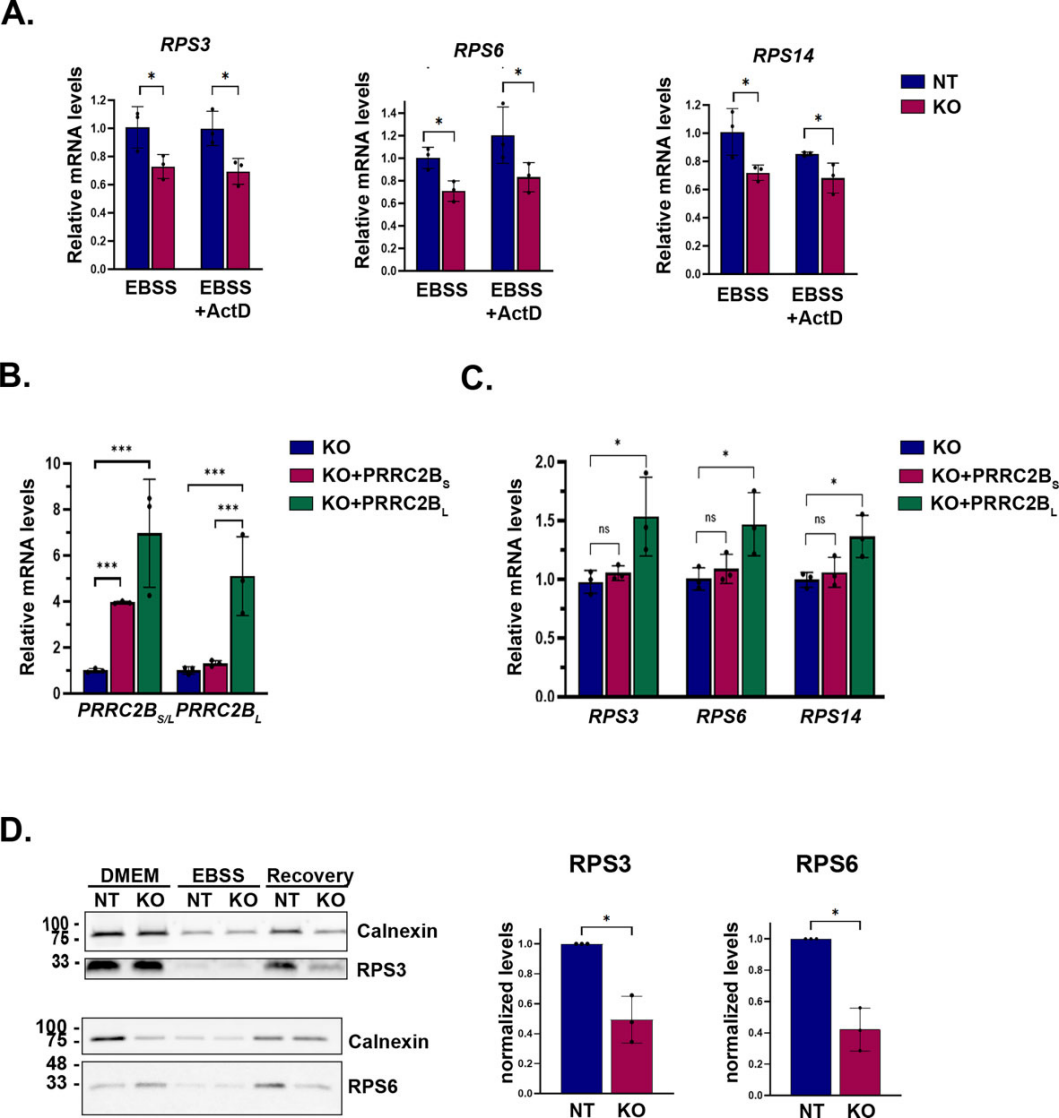
PRRC2B_L_’s maintenance of TOP mRNA stability during starvation is necessary for the resumption of translation of ribosomal proteins during recovery. (**A**) qRT-PCR showing levels of *RP* mRNAs, as indicated, in NT or PRRC2B KO cells subjected to 24 h starvation in EBSS media in the presence or absence of 50 nM Actinomycin D (ActD). mRNA levels are reported as fold-change for each gene, normalized to mean levels in NT samples in EBBS media. The means ± SD of three independent biological experiments are shown, with statistical significance determined by two-way ANOVA with Sidak’s post-hoc multiple comparison test. *, *P *< 0.05. Note that comparisons between EBSS and EBSS + ActD in either NT or KO conditions were not significant. (**B**) qRT-PCR showing levels of *PRRC2B* in PRRC2B KO cells transfected with either empty pCDNA3, PRRC2B_L_-HA or PRRC2B_S_-HA, and subjected to 48 h starvation in EBSS media. Primers used recognize either both isoforms (PRRC2B_L/S_) or only the long isoform (PRRC2B_L_). mRNA levels are reported as fold-induction for each primer set, normalized to mean levels in control KO samples. The means ± SD of three independent biological experiments are shown, with statistical significance determined by one-way ANOVA with Tukey’s post-hoc comparison test on the corresponding ΔΔCt values ***, *P *< 0.0005. (**C**) qRT-PCR showing levels of *RPS3, RPS6*, or *RPS14* as in (**B**). mRNA levels are reported as fold-change for each primer set, normalized to mean levels in KO samples. The means ± SD of three independent biological experiments are shown, with statistical significance determined by one-way ANOVA with Tukey’s post-hoc comparison test on the corresponding ΔΔCt values *, *P *< 0.05. (**D**) PRRC2B KO and NT control cells under normal growth conditions, following 48-h starvation (EBSS) and after 1-h restoration of normal growth media (Recovery), were incubated with Met analogue AHA, which was then covalently attached to biotin by Click chemistry. Biotin-labeled nascent proteins were purified from total cell lysates on avidin beads and subjected to western blot with antibodies to RPS3 and RPS6, or calnexin, as a non-TOP loading control. Shown are representative blots, with graphs summarizing the data from three independent biological repeats. RPS levels during recovery were quantitated by densitometry and normalized to calnexin levels. Within each experiment, RPS protein values in control NT lysates were set as 1, and the matched KO samples were expressed as a ratio of NT. Data from the three experiments are plotted as individual data points with mean ± SD. Statistical significance was determined by 1-sample *t*-tests with a hypothetical mean of 1; *, *P *< 0.05.

We next explored the mechanism by which PRRC2B KO affects TOP mRNA levels during starvation. Specifically, we tested whether PRRC2B regulates TOP mRNA transcript levels during starvation by indirectly modulating transcription of *RP* genes. To this end, we performed qRT-PCR during starvation to examine the effect of PRRC2B perturbations on *RP* transcript levels under conditions of transcriptional inhibition by Actinomycin D (ActD). *RPS3, RPS6*, and *RPS14* served as representative TOP genes. *DDIT3 (CHOP)* served as a control gene that is specifically transcriptionally induced during amino acid deprivation [58]. First, ActD treatment did not have any significant effect on *RP* transcript levels during starvation (Fig. 6A), indicating that these genes are not transcriptionally regulated under these conditions. This is consistent with the suppression of global transcription in general, and *RP* genes in particular, that occurs upon amino acid deprivation [59–61]. In contrast, as expected, ActD treatment greatly reduced the starvation-induced expression of *DDIT3* (Supplementary Fig. S5A). KO of PRRC2B led to significant reductions in *RPS3, RPS6*, and *RPS14* mRNA levels following nutrient starvation (Fig. 6A), independently confirming the RNA-seq transcriptomic data. Most importantly, ActD treatment did not prevent these reductions (Fig. 6A). This proves that PRRC2B’s effects on TOP mRNA levels occur independently of transcription. In contrast, PRRC2B KO had no effect on *DDIT3* levels following starvation, neither in the presence nor absence of ActD (Supplementary Fig. S5A). Thus, PRRC2B KO’s effects on *RP* mRNA levels are not due to modulation of *RP* gene transcription during nutrient deprivation.

As TOP mRNA levels following mTOR inhibition are maintained due to LARP1-dependent stabilization mechanisms, we hypothesized that the KO of PRRC2B perturbed this process. Accordingly, PRRC2B_L_, but not the short isoform that does not interact with LARP1, should be involved. Since the KO reduced levels of both isoforms, we ectopically expressed PRRC2B_L_ or PRRC2B_S_ in PRRC2B KO cells and examined whether either could rescue the stabilization of TOP mRNA in EBSS conditions. qRT-PCR using primers recognizing both isoforms or only the long isoform confirmed the expression of PRRC2B_L_ and/or PRRC2B_S_ in the transfected KO cells (Fig. 6B). Examination of western blots of lysates prepared in parallel confirmed the depletion of both isoforms and their restoration by ectopic expression at the protein level (Supplemental Fig. S5B). It should be noted that PRRC2B_S_ was actually expressed at higher levels than its endogenous counterpart. Notably, only the expression of PRRC2B_L_ significantly increased levels of *RPS3, RPS6*, and *RPS14* mRNA compared to the control KO (Fig. 6C). The fact that PRRC2B_S_ did not rescue PRRC2B KO’s effect on mRNA maintenance, despite its greater expression relative to endogenous protein, indicates that it is the KO of PRRC2B_L_ specifically that leads to reduced TOP mRNA levels during starvation.

### PRRC2B is required for immediate translation of ribosomal proteins during post-starvation recovery

The persistence of mRNAs encoding ribosomal proteins during prolonged amino acid starvation enables their immediate translation following re-feeding as they are prepared for ribosomal loading [28]. Thus, by impairing this persistence, PRRC2B KO should also affect the first wave of translation of these mRNAs upon nutrient restoration. To assess this, we measured PRRC2B KO’s effects on *de novo* synthesis of ribosomal proteins during basal, amino acid starvation (EBSS), and nutrient restoration (re-feeding) conditions. Nascent proteins of NT and PRRC2B KO HEK 293T cells were labeled with AHA, a Met analogue that can be isolated and detected via a biotin moiety that is subsequently attached by Click chemistry. To assess translation of specific proteins, labeled proteins were isolated using avidin beads and subjected to western blotting with antibodies to RPS3 and RPS6, as representative TOP mRNA-encoded proteins, and calnexin, a non-TOP protein (Fig. 6D). *De novo* synthesis of both TOP and non-TOP proteins was greatly reduced following 48 h of amino acid starvation. The latter reflects global mechanisms that reduce cap-dependent translation in response to mTOR inhibition (e.g. 4EBP dephosphorylation) [3], while the reduction of RPS translation resulted also from the additional suppressive mechanism imposed by LARP1 dephosphorylation [44, 47, 49, 50, 51, 62]. PRRC2B KO’s effect on basal translation was variable and not reproducible, and did not affect translation repression during starvation. Resynthesis of all proteins was observed 1 h after restoration of nutrients, with the ribosomal proteins showing the most robust recovery, consistent with their prioritized translation [53]. Most importantly, the *de novo* synthesis of RPS3 and RPS6 proteins following re-feeding was reduced in PRRC2B KO cells (Fig. 6D, Supplementary Fig. S5C). Thus, PRRC2B is required for the immediate translation of TOP mRNAs such as RPS3 and RPS6 following restoration of nutrients, indicating the functional significance of its contribution to preserving TOP mRNA levels during nutrient starvation.

## Discussion

Here, we identify two expressed isoforms of the RNA-binding protein PRRC2B: a long form, PRRC2B_L_, and a shorter form, PRRC2B_S_, lacking the middle region of the protein. Our data indicate that both PRRC2B_L_ and PRRC2B_S_ bind multiple translation initiation factors, and interact with and co-fractionate with the 40S ribosome. The common N- and C-termini that are shared by both proteins have been shown to be important for protein binding; the N-terminus in particular has been shown to bind translation initiation factors [19, 20]. We confirm the role of the N-terminal BAT2 domain in interacting with both canonical and non-canonical translation initiation factors, and show that this characteristic is common to both PRRC2B isoforms. By assessing the effect of RNase treatment on the presence of proteins in these co-IPs, we distinguished between direct and indirect protein-protein interactions. For example, while EIF3D and RPS6 interact with PRRC2B even in the presence of RNase, EIF4G2’s interaction with PRRC2B was strongly suppressed, suggesting that the presence of RNA enables or stabilizes the interaction. Interestingly, both long and short isoforms are diverted to stress granules following arsenic stress. As only PRRC2B_L_ interacts with stress granule assembly proteins such as FMR1, G3BP1, and G3BP2, the translocation of PRRC2Bs presumably results from its association with stalled translation initiation complexes that accumulate in the stress granules. Based on the similar behavior demonstrated for PRRC2B_L_ and PRRC2B_S_ in binding translation initiation factors and the 40S ribosome, we predict that both isoforms function as translation initiation regulators, with PRRC2B_S_ playing a minor role due to its lower expression levels in the cell lines assayed and its reduced affinity for its interacting partners.

The middle RG-rich exon 16 that is unique to PRRC2B_L_ confers distinct functional properties to the long isoform, resulting in different protein interactomes and mRNA binding abilities. The middle portion of PRRC2B (residues 750–1500) was shown previously to bind RNA [20]. This region was likewise shown to be involved in mRNA binding and translation activity in the *Drosophila* paralog Nocte [19]. It is presumably the RG-rich domain of this region that mediates RNA binding. It has previously been proposed that the Gly residues permit flexibility of the protein backbone, which enables conformational plasticity of the disordered region. The positively charged Arg residues, on the other hand, provide non-specific electrostatic interactions with the mRNA and also more specific hydrogen bonding with mRNA tertiary structures [63]. Thus, overall, the RG-dipeptide motifs enhance the interaction with mRNAs. The RER motif following the N-terminal BAT2 domain may also potentially mediate RNA interactions. This is consistent with RNA-binding properties of intrinsically disordered RBPs that lack the classical RNA-binding domains, such as K-homology domains, Zinc fingers, and RNA Recognition Motifs (RRM), and instead contain multiple RNA interaction sites of low complexity [5, 64]. The multiple sites combined with the lack of an established, stable structure may enable greater adaptability and specificity in substrate recognition. Consistent with this hypothesis, we showed that unlike PRRC2B_S_, which lacks the RG-rich domain, PRRC2B_L_ specifically interacts with 5′ TOP mRNAs.

The middle domain of PRRC2B_L_ is also responsible for the preferential interaction of PRRC2B_L_ with two major groups of proteins: the mitochondrial small ribosome subunit and regulatory RNA-binding proteins. The interaction with multiple protein components of the small subunit of the mitochondrial ribosome is intriguing, as PRRC2B_L_ does not localize to the mitochondrial matrix, where mitochondrial translation occurs. Furthermore, and in contrast to translation initiation factors of the cytosol that strongly interacted with PRRC2B, mitochondrial initiation factors were not part of the PRRC2B interactome. Therefore, PRRC2B is unlikely to play a parallel role in translation initiation of mitochondrial-encoded mRNAs within the mitochondria. Presumably, the interaction occurs in the cytosol. The presence of MRPSs within the PRRC2B_L_ interactome may reflect a potential interaction that occurs when the former accumulate in the cytosol, such as during import failure or mitochondrial stress and rupture. Our current study does not address the mechanistic and functional significance of PRRC2B interactions with MRPSs, and additional investigation is required.

We have shown that at least some of the proteins within the second group of RPBs, such as LARP1 and PABP, require the presence of the RG-rich region for efficient interaction. Moreover, treatment with RNase reduced the interaction, implying that these protein-protein interactions depend on PRRC2B’s interaction with RNA. It is possible that the interaction is indirect, i.e. both proteins simultaneously bind the same mRNA, e.g. a TOP mRNA, which brings the 2 proteins into the same RNA-protein complex from which they co-IP. Alternatively, binding to mRNA may induce refolding of the intrinsically disordered PRRC2B, as such disordered proteins can become structured upon binding to other proteins or DNA/RNA [41], and in its newly acquired structured form, the protein gains the ability to bind its interacting partner. Each of these two models can accommodate the possibility of functional interactions between these RNA-binding proteins in a given RNA-protein complex.

PRRC2B_L_’s specific interaction with LARP1 and PABP proteins, and likewise, mRNAs encoding *RPS* proteins, suggests a novel function for the protein in regulating TOP mRNAs during starvation. This function distinguishes the long and short isoforms and is consistent with our observation that the ratio between the two isoforms shifts even further in favor of PRRC2B_L_ during starvation. Under basal conditions, when mTOR is active, TOP mRNAs are efficiently translated, and LARP1 binds to the 3′UTR of these mRNAs together with PABPC1 through its N-terminal La and PAM2 domains [45, 65]. Upon the inhibition or inactivation of mTOR, such as following amino acid starvation, translation of TOP mRNAs is attenuated, due to two mechanisms that prevent cap-dependent initiation: dephosphorylation of mTOR substrate EIF4EBP, which then binds and sequesters the cap-binding EIF4E, and dephosphorylation of LARP1, which allows it to also interact with the TOP motif and the m(7)G 5′cap through its C-terminal DM15 domain, thereby blocking EIF4G1 and EIF4E binding [48–51, 62]. While the former mechanism is general and affects all EIF4E/cap-dependent cellular mRNAs, the latter is distinct for TOP mRNAs, providing a means for mTOR to specifically regulate the translation-related proteome. Yet, more recent data have shown that the 4EBP mechanism is the dominant means of regulating TOP mRNAs following mTOR inhibition [52]. According to this, LARP1’s primary function in these stress conditions is to maintain and stabilize TOP mRNA levels so that a pool is immediately available for ribosome loading and translation when metabolism is restored [28]. Several non-mutually exclusive mechanisms have been proposed in this context, which involve binding of LARP1 to the polyA tail in conjunction with PABPC1, promoting post-transcription polyA elongation, preventing de-adenylation, and blocking degradation [28, 45, 46, 52–54]. LARP1-bound TOP mRNAs are sequestered in a complex with the 40S or non-translating 80S ribosomes [53, 54]. LARP1 directly binds 40S subunits in a manner that blocks its mRNA channel, thereby preventing translation even upon recruitment of the 60S subunit [55]. In any event, upon re-feeding and recovery of mTORC1 activity, LARP1 is immediately phosphorylated by mTOR, and its release from the 5′ UTR of TOP mRNAs, in conjunction with multi-phosphorylation and inactivation of 4EBP, enables their translation by cap-dependent factors, and also serves to recruit mTORC1 to the 3′UTR to promote translation initiation [57].

We have shown that PRRC2B_L_ not only binds to TOP mRNAs *RPS3* and *RPS6*, but moreover, is necessary for maintaining levels of TOP mRNAs in general, and particularly ribosomal protein mRNA, during prolonged amino acid and serum starvation. This occurs independently of any transcriptional effects, which is generally attenuated during starvation and thus minimally contributes to *RP* expression [59, 61]. PRRC2B_L_ is therefore presumed to contribute to TOP mRNA stability mechanisms, which have extensively been established in the context of its known regulators, LARP1 and PABP, as detailed above. Our data does not support a role for PRRC2B in LARP1’s function as an inhibitor of TOP mRNA translation during starvation and mTOR inhibition [44, 49, 57, 62, 66]. We expect that the attenuation of TOP mRNA maintenance during the starvation stage that is observed in the PRRC2B KO, and the resulting attenuation in ribosomal protein synthesis during recovery, would likewise delay the recovery of ribosomal biogenesis, and as a consequence, the increase in global protein synthesis that occurs during metabolic shift. Thus, in addition to its previously demonstrated role in translation initiation under basal conditions [18, 20], PRRC2B likely *indirectly* affects translation by regulating levels of ribosomal protein biogenesis following stress. We cannot exclude the possibility, however, that PRRC2B’s effect on ribosomal protein synthesis stems also from its role as a translation initiator and ability to recruit the ribosome to TOP mRNAs, ensuring rapid recovery of proteins critical for further translation even prior to full re-phosphorylation of EIF4EBP and release of EIF4E for binding to the 5′ mRNA cap. Whether PRRC2B contributes directly to translation initiation of TOP mRNAs, in addition to its role in their preservation, remains to be determined.

Thus, we have identified a new functional role for PRRC2B, residing in the long isoform, in binding and maintaining TOP mRNA levels during starvation, independent of its translation-initiation functions. This TOP modulating function is uniquely mediated by PRRC2B_L_. The introduction of an additional RBP that regulates TOP mRNA sheds light on previously described LARP1-dependent mechanisms.

## Supplementary Material

gkaf1334_Supplemental_Files

## Data Availability

The mass spectrometry proteomics data are available in the ProteomeXchange Consortium via the PRIDE [67, 68] partner repository (http://www.ebi.ac.uk/pride) and can be accessed with the dataset identifiers PXD057527 (IP-MS) and PXD057790 (modification analysis). The RNA-seq datasets generated in the current study are available in the Gene Expression Omnibus (GEO, https://www.ncbi.nlm.nih.gov/geo/query/acc.cgi?acc=GSE282113) and can be accessed with accession ID GSE282113. All other data underlying this article are available in the article and in its online supplementary material.
