# Supplementary material for: The RNA-binding protein PRRC2B preserves 5′ TOP mRNA during starvation to maintain ribosome biogenesis during nutrient recovery
Source: Nucleic Acids Res. 2025 Dec 12;53(22):gkaf1334. doi: 10.1093/nar/gkaf1334 (PMC12700102; doi:10.1093/nar/gkaf1334)
Supplement: gkaf1334_Supplemental_Files [file gkaf1334_supplemental_files.zip › Goldberg_et_al_Supplementary_materials.pdf]

Supplementary Materials for

The RNA-binding protein PRRC2B preserves 5' TOP mRNA during starvation to maintain ribosome biogenesis during nutrient recovery

Nadav Goldberg *et al.*

*Corresponding author. adi.kimchi@weizmann.ac.il

This PDF file includes:

Supplementary Figs. S1 to S5
Supplementary Tables S1-S5 captions

Other Supplementary Materials for this manuscript include the following:

Supplementary Tables: S1-S5 (single excel file with multiple tabs)

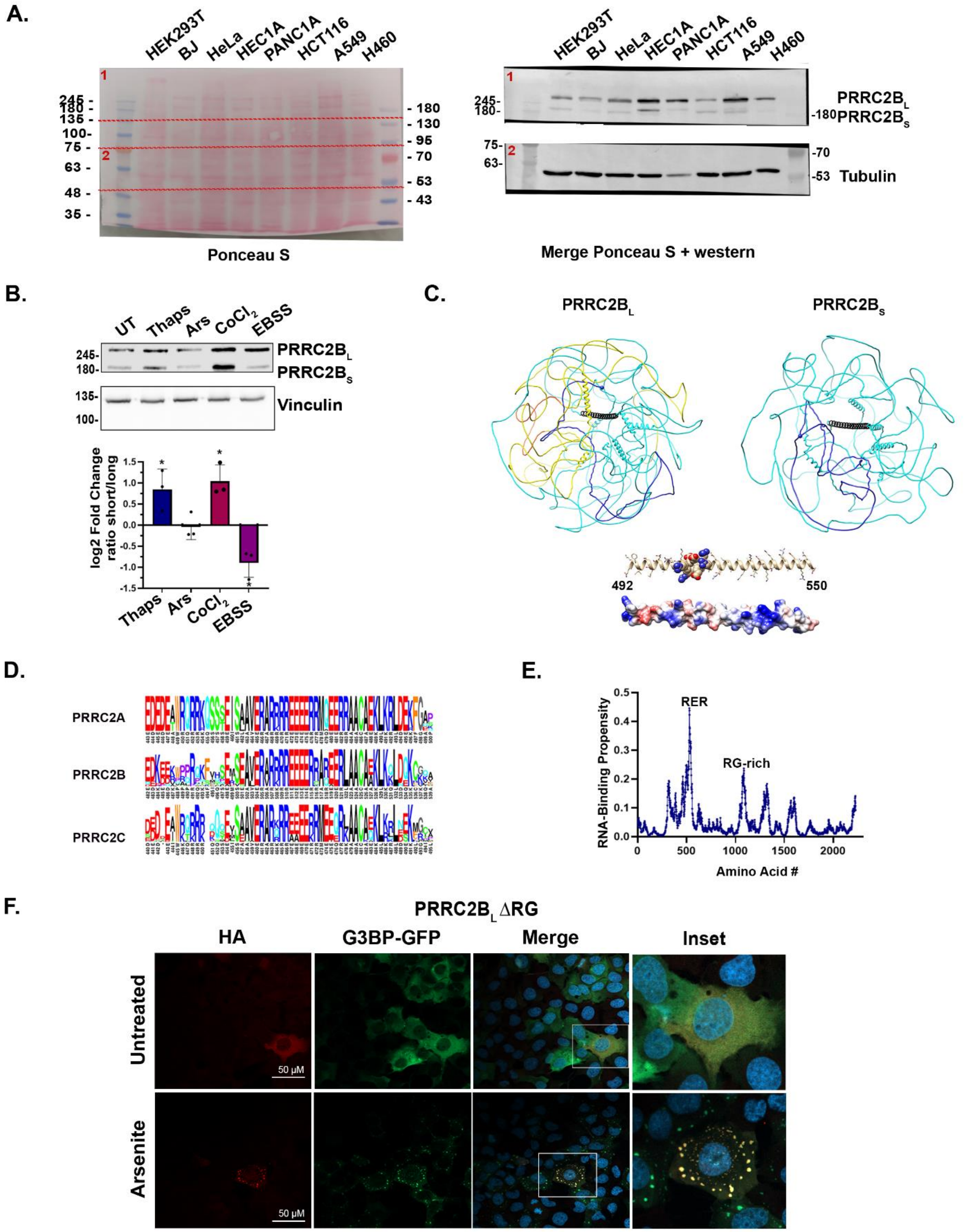

Supplementary Figure S1.

Additional analysis of PRRC2B isoforms. (**A**) Full Ponceau S stained membrane of western blot shown in Figure 1A (left) and image of strips 1 and 2 probed with indicated antibodies, merged on top of the

Ponceaus staining to visualize MW markers. (**B**) Levels of PRCC2B isoforms in different cellular stress conditions. HEK 293T cells were left untreated (UT), or treated with 1 µM thapsigargin for 4 h to induce ER stress, 100 µM sodium arsenite for 4 h, 500 µM $CoCl_2$ for 4 h to mimic hypoxic conditions, or starved of amino acids and serum in EBSS media for 48 h. Cell lysates were subjected to Western blots for endogenous PRRC2B or Vinculin, as a loading control. Shown in a representative western blot from 1 of 3 experiments. Quantities of $PRRC2B_L$ and $PRRC2B_S$ were determined by densitometry. Graph shows the fold-change in the ratio of $PRRC2B_S$ to $PRRC2B_L$ in each condition compared to untreated cells, expressed as mean±SD of log2 transformed values (log2 UT=0), with individual data points from n=3 experiments. Statistical significance vs. untreated was determined by one-way ANOVA with Dunnett's post-hoc correction; * $p<0.05$. (**C**) Three-dimensional model structures of $PRRC2B_L$ (left) and $PRRC2B_S$ (right) according to predictions by Alphafold-3 (AF-3) server. AF-3 produced 5 models for each isoform with low Predicted Template Modelling (PTM) scores: 0.2 and 0.18 for the long and short variants, respectively. However, very high per-atom confidence (pLDDT>90) is predicted for a short segment of PRRC2B, residues 502-542, which forms a long helix (shown in black in the 2 structures). The predicted confidence of the edges of this helix, residues 495-501 and 543-550, is lower (pLDDT>70). The pLDDT range for the backbone atoms of residues 495-550 in the long variant of PRRC2B is 59.7-96.9, averaging to 90.5±7.7. For the short variant of PRRC2B the pLDDT range for the backbone atoms of residues 495-550 is 76.1-98.0, averaging to 94.6±4.4. Very similar results are provided for the four additional AF-3 models in each prediction and for models produced with different seeds. The top model for each isoform are shown recolored in cyan with the BAT2 domain (1-200) in blue. The N-terminus is indicated by the blue sphere. The region encoded by exon 16 that is lacking in the short isoform is colored yellow, with the RG-rich region 1061-1124 in red in the structure of $PRRC2B_L$. The long high confidence helix 492-550 is also presented in the middle of the two structures as a ball-and-stick model with oxygen and nitrogen atoms in red and blue, respectively, and as a solvent accessible surface colored by the Coulombic potential, with blue indicating positive, and red indicating negative surface regions, and white for neutral regions. Note that the highly conserved BAT2 segment is unstructured and does not form a folded domain. (**D**) Multiple sequence alignment within the RER domain for all PRRC2 family members, PRRC2A, B, and C, shown according to the human amino acid position numbers. (**E**) Prediction of RNA binding capacity of PRRC2B according to DisoRDPbind. Each residue is assigned a value between 0 and 1 representing its likelihood to be involved in disordered RNA binding. Peaks correspond to regions likely to bind mRNA. Positions of RER and RG-rich domain are indicated. (**F**) U2OS cells stably expressing GFP-G3BP were transfected with HA-tagged $PRRC2B_L$ deleted of the RG-rich domain and treated with sodium arsenite or left untreated for 30 min. Cells were fixed and stained with anti-HA and DAPI to detect nuclei. Insets at far right are enlargements of the boxed regions within the corresponding Merge panels. Shown are representatives of 8-10 fields examined. (Related to Fig. 1,2)

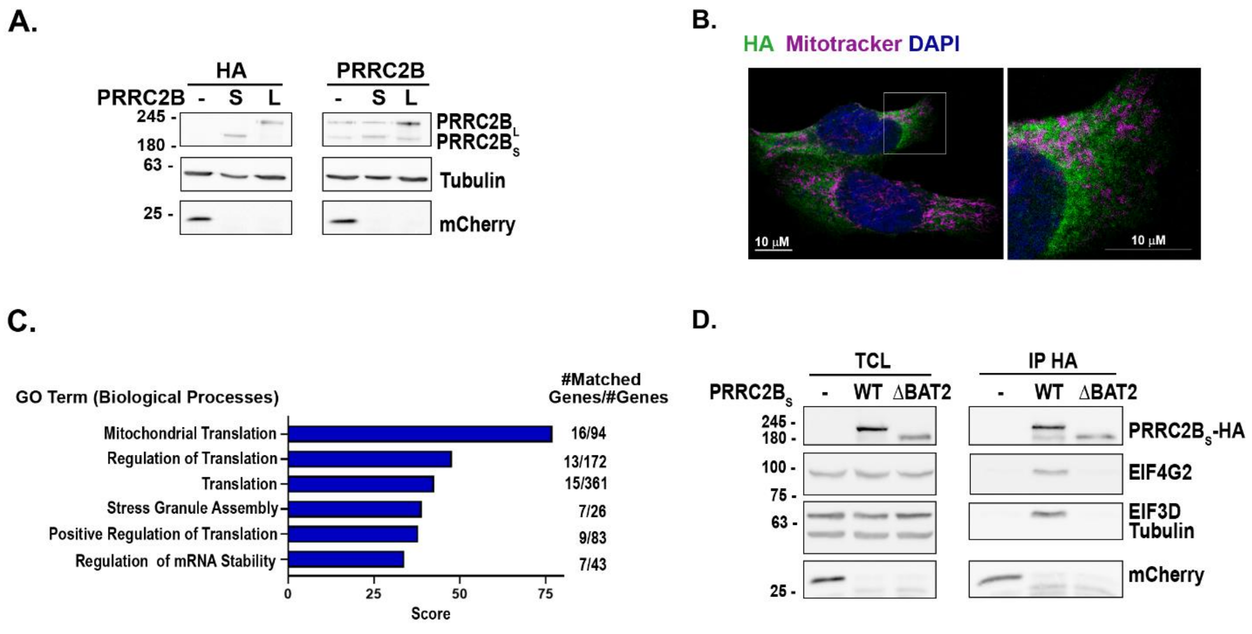

Supplementary Figure S2.
Additional analysis of PRRC2B$_L$ and PRRC2B$_S$ interactomes and localization. (**A**) Representative western blot probed with either antibodies to HA (recognizing exogenous PRRC2B only) or PRRC2B (recognizing both exogenous and endogenous proteins) showing expression levels of HA-tagged mCherry, PRRC2B$_L$ and PRRC2B$_S$ in HEK 293T cells under same conditions as used for IP-Mass Spec experiment. (**B**) HeLa cells transiently transfected with pcDNA plasmid expressing PRRC2B$_L$-HA were stained with anti-HA antibodies, Mitotracker to detect mitochondria and DAPI for nuclei. Middle panel is enlargement of boxed area in left panel. No overlap between HA and Mitotracker signals is observed. (**C**) Top 6 significant GO terms (Biological Processes) and scores identified by GeneAnalytics analysis of the set of 69 proteins that preferentially interact with PRRC2B$_L$. The number of matched genes within the set out of the total number of genes in the particular biological process is indicated at right of graph. (**D**) HEK 293T cells transiently transfected with pcDNA plasmids expressing HA-Cherry, tagged PRRC2B$_S$-HA full length or ΔBAT2 mutant were immunoprecipitated with anti-HA antibodies, and lysates and IPs subjected to western blot analysis for the indicated proteins. Shown is a representative blot of 5 independent experiments. (Related to Fig. 3)

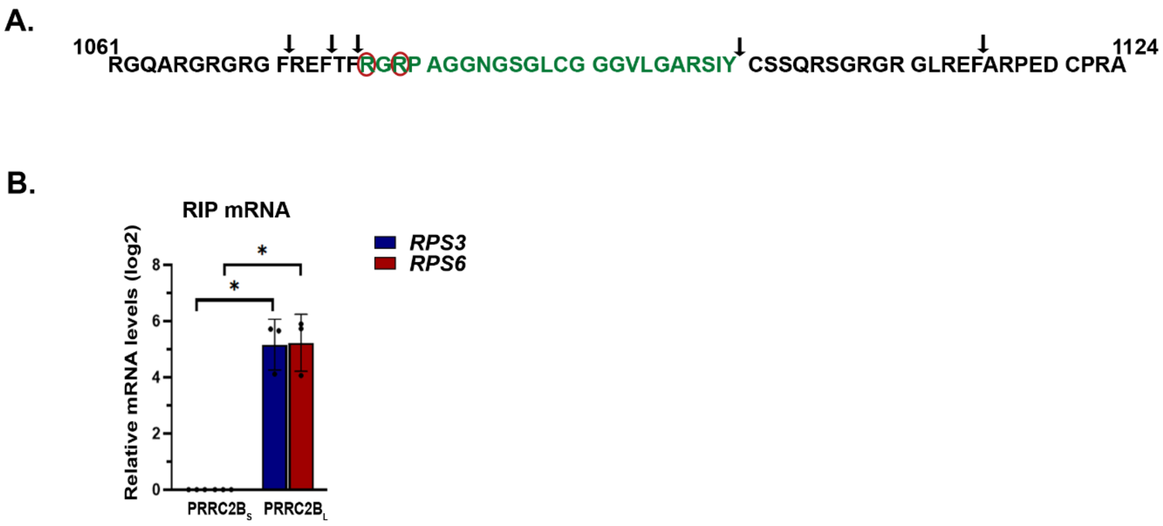

Supplementary Figure S3.

Additional analysis of unique features of PRRC2B$_L$. (**A**) Sequence of RG-rich domain indicating Arg residues identified as methylated by Mass Spec following chymotrypsin digestion of the protein, based on data shown in Table S2. Peptide identified by Mass Spec is colored green, remaining peptides were not detected. Chymotrypsin cleavage sites are indicated by arrows, methylated residues are circled in red. (**B**) RIP assay. HEK293T cells were transfected with either mCherry-HA, PRRC2B$_L$-HA or PRRC2B$_S$-HA, starved for 48 h in EBSS media, and lysates were immunoprecipitated with anti-HA antibody-bound beads. qRT-PCR for RPS3 and RPS6 was performed on mRNA that co-IPed with HA-tagged proteins (RIP mRNA). Graph shows relative mRNA levels, expressed as mean±SD with individual data points of n=3 independent biological experiments. mRNA fold-changes were calculated by comparing to levels in the mCherry IP (ΔCt), and then corrected for any differences in protein levels of each isoform present in the individual IP. In order to additionally correct for the inherent variability in IP efficiency among the experiments, the relative fold-change was normalized to PRRC2B$_S$ IP levels in each experiment, which was set at 1, and the values were then log2 transformed. Statistical significance was determined by one-sample T test with hypothetical mean of 0, * $p<0.05$. (Related to Fig. 4)

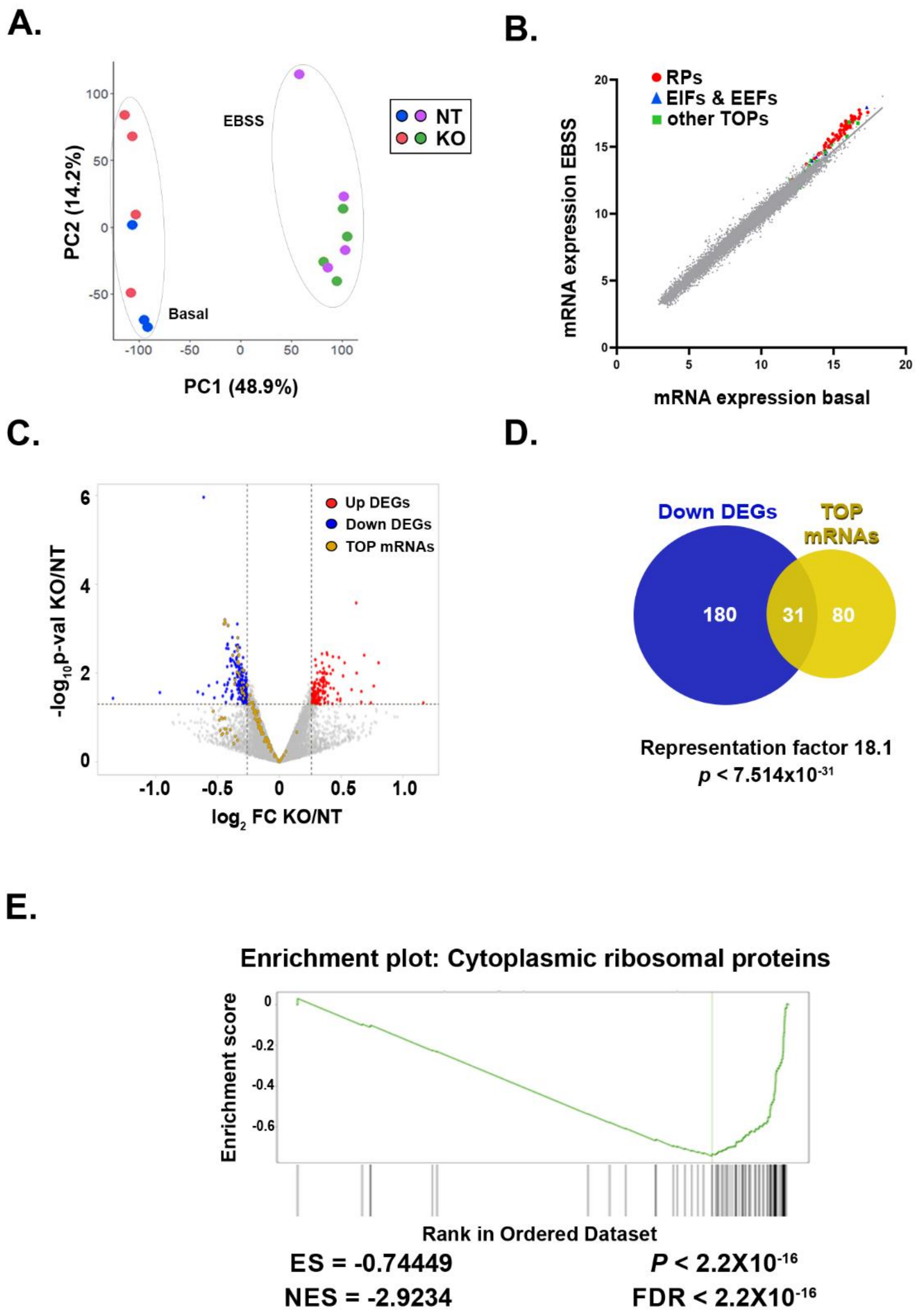

Supplementary Figure S4.

Additional analysis of RNA-seq data in starved PRRC2B cells. (**A**) Principle Component Analysis (PCA) of RNA seq data from NT or PRRC2B KO, basal or EBSS conditions. (**B**) Normalized expression levels of total mRNAs under basal conditions vs starvation (EBSS), for protein-coding genes only in NT control cells. mRNAs above the grey line are expressed at higher relative levels during starvation compared to the rest.

TOP mRNAs are highlighted, specifically ribosomal proteins and eukaryotic initiation and elongation translation factors (EIFs, EEFs). (**C**) Volcano plot of the fold-change in mRNA expression levels in PRRC2B KO vs NT control under starvation conditions, vs. their significance expressed as $-\log_{10}$ *p*-value. Increased and decreased differentially expressed genes are indicated in red and blue, respectively. Yellow dots represent mRNAs encoding TOP mRNAs. (**D**) Venn diagram showing overlap between TOP mRNAs and the group of statistically significant down-regulated genes (down DEGs). (**E**) GSEA enrichment plot of the Cytoplasmic Ribosomal Proteins set (87 genes), ranked by fold change from highest to lowest. The running enrichment score (green line) demonstrates a significant negative enrichment, with a normalized enrichment score (NES) of –2.9234. Vertical black bars indicate the positions of individual analyte set genes in the ranked list, with 70 genes contributing to the leading edge. (Related to Fig. 5.)

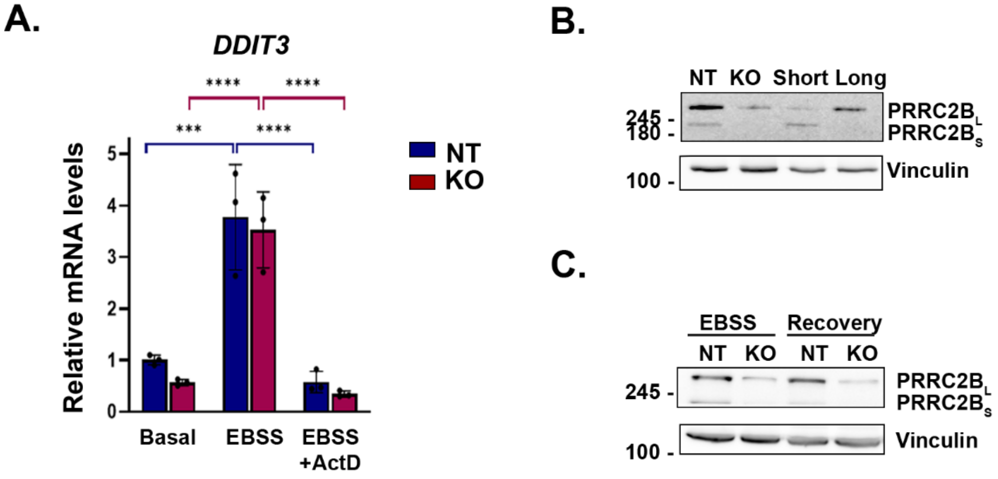

Supplementary Figure S5.

Additional analysis of PRRC2B's function in maintaining TOP mRNA levels during starvation. (**A**) qRT-PCR showing mRNA levels of *DDIT3* in control NT or PRRC2B KO HEK 293T cells subjected to basal growth conditions, or 24 h starvation in EBSS media with or without the addition of 50 nM actinomycin D (ActD) to inhibit transcription. Shown are means and individual data points from n=3 biological experiments, normalized to mean levels in basal conditions for NT. Statistical significance was determined by 2-way ANOVA with Sidak's post-hoc multiple comparisons test. ***, $p < 0.005$; ****, $p < 0.0001$; comparisons between NT and KO within each growth condition, or between Basal and EBSS+ActD conditions were not statistically significant. (**B**) Western blot from one representative experiment showing levels of PRRC2B isoforms in NT cells or PRRC2B KO cells transfected with either empty pCDNA3, $PRRC2B_L$-HA or $PRRC2B_S$-HA, and subjected to 48 h starvation in EBSS media. Vinculin was used as a loading control. (**C**) Western blot from one representative experiment showing levels of PRRC2B isoforms in NT cells or PRRC2B KO cells subjected to 48 h starvation in EBSS media and during subsequent recovery following restoration of nutrients in normal growth media. Vinculin was used as a loading control. (Related to Fig. 6)

Supplementary Table S1.

PRRC2B interactome (IP-Mass-Spec data).

mCherry-HA, PRRC2B$_L$-HA and PRRC2B$_S$-HA were immunoprecipitated from HEK293T cells (n=4) and IPs were subjected to LC-MS. First set of columns (shaded orange) shows proteins with significant increased abundance (≥2 unique peptides, FC>2, FDR< 0.05) in the PRRC2B$_L$ IP compared to control mCherry-. Second set of columns (green shaded) shows proteins with significant increased abundance (≥2 unique peptides, FC>2, FDR< 0.05) in the PRRC2B$_S$ IP compared to control mCherry-HA. Third set of columns (yellow shaded) shows comparison of abundance of interacting proteins in the PRRC2B$_L$ IP vs the PRRC2B$_S$ IP, listing those with preferential interaction with the PRRC2B$_L$ (FC>2, p< 0.05). Proteins are listed in order of most abundant.

Supplementary Table S2.

Methylation of PRRC2B peptides (Mass-Spec protein modification data)

Chymotrypsin-cleaved peptides identified by LC-MS of PRRC2B$_L$-HA were analyzed for protein post-translational modifications. Data indicate PRRC2B$_L$ peptide sequences and methylations found within each peptide.

Supplementary Table S3.

Gene Expression during prolonged nutrient starvation (RNA-seq data)

First set of columns shows the complete dataset (basemean>10) for comparison of control NT samples in basal vs EBSS conditions. The set of up-regulated DEGs (FC>1.2, p<0.05) are shown in the second set of columns (shaded yellow).

Supplementary Table S4.

Gene Expression during prolonged nutrient starvation upon PRRC2B KO (RNA-seq data)

First set of columns shows the complete dataset for comparison of control NT and PRRC2B KO samples in EBSS conditions. The set of down- and up-regulated DEGs (FC>1.2, p<0.05) are shown in the second and third set of columns, shaded pink and yellow, respectively. Lists of DEGs are divided into coding genes and non-coding genes. Genes highlighted have been previously classified as TOP genes by either (*28*) or (*29*); red, ribosomal proteins, blue, translation initiation or elongation factors, green, all other TOP genes. Note that 111 TOP genes were identified in the dataset.

Supplementary Table S5.

Primers used for quantitative Real Time-PCR

List of forward (F) primers, and reverse (R) primers used for analysis of indicated genes.
